# All-Solution Processed Single-Layer WOLEDs Using [Pt(salicylidenes)] as Guests in a PFO Matrix

**DOI:** 10.3390/nano12142497

**Published:** 2022-07-20

**Authors:** José Carlos Germino, Luís Gustavo Teixeira Alves Duarte, Rodrigo Araújo Mendes, Marcelo Meira Faleiros, Andreia de Morais, Jilian Nei de Freitas, Luiz Pereira, Teresa Dib Zambon Atvars

**Affiliations:** 1 Chemistry Institute, University of Campinas—UNICAMP, Campinas 13083-862, Brazil; lg.alvesduarte@gmail.com (L.G.T.A.D.); mmfaleiros@gmail.com (M.M.F.); tatvars@unicamp.br (T.D.Z.A.); 2 Department of Physics and i3N—Institute for Nanostructures, Nanomodelling and Nanofabrication, University of Aveiro, 3810-193 Aveiro, Portugal; 3 São Carlos Institute of Chemistry, University of São Paulo—USP, São Carlos 13566-590, Brazil; rodrigoaramendes@gmail.com; 4 Center for Information Technology Renato Archer—CTI, Campinas 13069-901, Brazil; andreiademoraes6@gmail.com (A.d.M.); jilian.freitas@cti.gov.br (J.N.d.F.)

**Keywords:** white-OLED, platinum(II) complexes, photoluminescence, solution-deposited devices

## Abstract

Herein, we report the synthesis and characterization of two Pt(II) coordination compounds, the new platinum(II)[*N*,*N*′-bis(salicylidene)-3,4-diaminobenzophenone)] ([Pt(sal-3,4-ben)]) and the already well-known platinum(II)[*N*,*N*′-bis(salicylidene)-o-phenylenediamine] ([Pt(salophen)]), along with their application as guests in a poly [9,9-dioctylfluorenyl-2,7-diyl] (PFO) conjugated polymer in all-solution processed single-layer white organic light-emitting diodes. Completely different performances were achieved: 2.2% and 15.3% of external quantum efficiencies; 2.8 cd A^−1^ and 12.1 cd A^−1^ of current efficiencies; and 3103 cd m^−2^ and 6224 cd m^−2^ of luminance for the [Pt(salophen)] and [Pt(sal-3,4-ben)] complexes, respectively. The Commission Internationale de l’Eclairage (CIE 1931) chromaticity color coordinates are (0.33, 0.33) for both 0.1% mol/mol Pt(II):PFO composites at between approximately 3.2 and 8 V. The optoelectronic properties of doped and neat PFO films have been investigated, using steady-state and time-resolved photoluminescence. Theoretical calculations at the level of relativistic density functional theory explained these results, based on the presence of the Pt(II) central ion’s phosphorescence emission, considering spin-orbit coupling relationships. The overall results are explained, taking into account the active layer morphological properties, along with the device’s electric balance and the emitter’s efficiencies, according to deep-trap space-charge models. Considering the very simple structure of the device and the ease of synthesis of such compounds, the developed framework can offer a good trade-off for solution-deposited white organic light-emitting diodes (WOLEDs), with further applications in the field of lighting and signage.

## 1. Introduction

Photo and electroluminescent coordination compounds are materials with several applications, including organic electronics [1,2,3,4,5]. Compared with inorganic materials, the chemical manipulation of these organic materials might create structures targeting specific properties or might even modulate those properties. Such outstanding attributes in terms of organic/organometallic chemistry are naturally of great interest [6]. In particular, the structural modification of conjugated polymers, coordination compounds, and molecules demonstrating delayed fluorescence [6,7,8,9,10,11,12] are good examples of this versatility for the purposes of organic or polymer light-emitting diodes (OLEDs), wherein colors might be tuned to the entire visible spectral range. Currently, one of the most important lines of research in these systems is the trade-off between device efficiency and structural simplicity.

Furthermore, different strategies are used to assemble organic LED devices with white emissions (WOLEDs) [13], which may include complex molecules with multiple chromophores [14,15], hybrid materials [13], excited-state intramolecular proton transfer (ESIPT) compounds [16,17,18], polymer blends with conjugated polymers [19,20], diodes with external color conversion layers (CCLs) [21,22], diodes with multilayers [23,24], thermally activated delayed fluorescent molecules [25,26], diode arrays with microlenses [27], nanocrystal/conducting polymer composites [28,29,30], and exciplex emitters [31]. The opportunities for innovation are truly huge.

Unlike fluorescent organic molecules that can only generate singlet excitons, which limits the maximum internal quantum efficiencies (IQE) to 25% (with the further consequence of a maximum theoretical quantum external efficiency—EQE—of only 5% [32]), coordination compounds containing heavy metals are able to produce both singlet and triplet excitons, due to their strong spin-orbit coupling, overcoming the spin-forbidden transition rule [33]; hence, in theory, they could reach a maximum IQE of 100% [34]. As a consequence, the synthesis of new coordination compounds containing heavy atoms with highly efficient phosphorescence emissions might be particularly interesting for applications in OLEDs [35,36,37,38]. Among the phosphorescent emitters in coordinated compounds, platinum (Pt(II)) complexes are of interest, due to their high photoluminescence quantum yields (PLQY) and wide range of emissions in the visible spectrum, which are easily modulated by the ligand framework [39,40,41,42,43,44,45,46,47,48].

In particular, salicylidene ligands are interesting due to the possibility of depicting ESIPT, leading to dual emission bands originating from the normal and tautomeric forms [16,17,49]; their Pt(II) compounds can achieve highly phosphorescent emissions due to the electronic transitions originating from their mixed-metal to ligand charge transfer (MLCT) and intra-ligand charge-transfer (ILCT) characteristics ([Pt(5d) → π *]) between the highest occupied and lowest unoccupied molecular orbitals (HOMO and LUMO, respectively) [50]. The possibility of forming triplet excitons is desirable for OLEDs since they might offer greater performance compared to materials forming singlet excitons [34]. In contrast to other Pt(II) coordination compounds [50], Pt(salicylidenes), or Pt(N_2_O_2_-Schiff bases) present an excellent trade-off between easy synthesizing and purification procedures to produce the ligand [16,17,51], as well as the Pt(II) complex itself [52], and the OLEDs’ figures of merit, mainly seen when we search for electroluminescent devices offering colors ranging from yellow to near-infrared emissions. Simple chemical substitutions can easily modulate these features on the salicylidene ligand framework, in order to achieve the different colors, presenting significant improvements to the charge-carrier transport characteristics, solubility, and, finally, excellent OLED performance under the wettable processing protocols. Moreover, the red emissions of such complexes can be mixed with a matrix blue emission, in two complementary colors, in order to achieve white emissions. In terms of possible lighting applications, the devices also need to be structurally simple; the fabrication process should be able to produce an emissive layer that is electrically well balanced, allowing a good trade-off between efficiency (which would be lower than a more complex device structure) and useful luminance.

**Figure 1:**
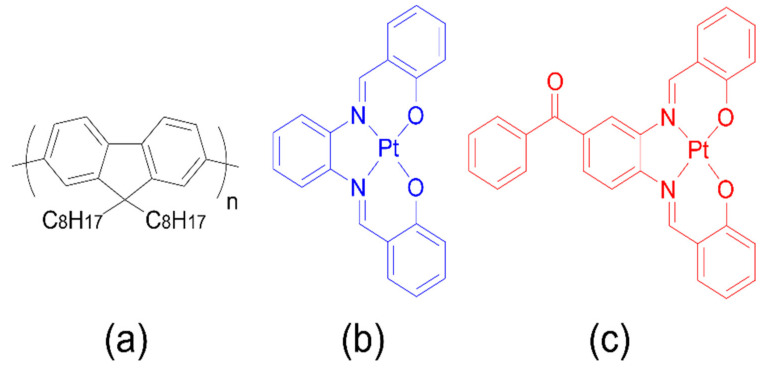
Molecular structures of PFO (**a**), [Pt(salophen)] (**b**) and [Pt(sal-3,4-ben)] (**c**) organic semiconducting materials.

In light of these considerations, herein we describe the synthesis and the structural and optical characterization of two Pt(II)salicylidene derivatives: [Pt(sal-3,4-ben)] and [Pt(salophen)] (Figure 1). We also study the performance of all-solution processed OLEDs with a simple structure: ITO/PEDOT:PSS/PVK/PFO:[Pt(salicylidenes)]/Ca/Al, with 0.1, 0.5, and 1.0% mol/mol (≈6 × 10^−4^ wt%, 3 × 10^−3^ wt% and 6 × 10^−3^ wt%, respectively) of the emitter in the polyfluorene matrix. The CIE1931 (*x,y*) coordinates achieved under the best white emission device tuning are (0.33, 0.33). The best results are obtained using a [Pt(sal-3,4-ben)] emitter with a maximum EQE at 15.3% (current and power efficiencies of 12.1 cd A^−1^ and 11.9 lm W^−1^, respectively) and luminance of over 6000 cd m^−2^. The most efficient device exhibits a high roll-off at the point of considerable operational luminance, in comparison to the turn-on voltage (V_on_) efficiency, but it maintains a low roll-off at the operational bias (EQE = 2% @ L = 100 cd m^−2^, EQE = 1.4% @ L = 1000 cd m^−2^, and EQE near 1.5% @ L = 5000 cd m^−2^). On the other hand, WOLEDs based on 0.1% of [Pt(salophen)] in a PFO matrix offer poor performance; however, they exhibit better functional roll-off. These results were established theoretically in terms of the electrical balance of the devices, involving charge mobilities, intrinsic defect formation, molecular and electronic structures, photophysical features, and the morphological characteristics. This approach was chosen to optimize a host: guest matrix, yielding particularly interesting results that can be further explored to improve solution-deposited OLEDs in the specific context of large-area printed electronic devices for lighting and signage applications. The framework developed is, therefore, a valuable contribution to that important field of research.

Finally, the combination of a PFO host matrix with Pt(II)salicylidenes presents the best choice of materials for achieving efficient solution-processed WOLED devices.

## 2. Results and Discussion

### 2.1. Structural Characterization

**Figure 2:**
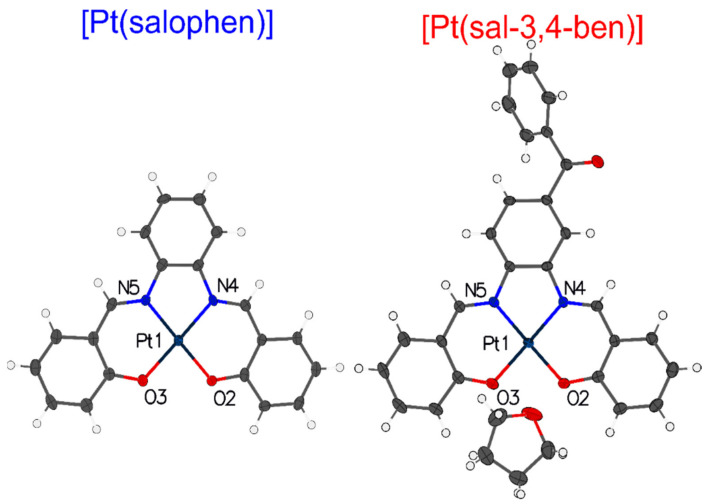
Molecular crystal structures of the [Pt(salophen)] and [Pt(sal-3,4-ben)] coordination compound ellipsoids, with 50% probability, where: hydrogen is the white balls; carbon is the gray ellipsoids; nitrogen is the blue ellipsoids; oxygen is the red ellipsoids; and platinum is the dark-blue ellipsoids.

Pt(II) coordination compounds were isolated from the reaction mixture by the addition of deionized water; then, they were dried and purified via a recrystallization process in DMF solvent, with addition of some THF drops until the solid solubilization (a simplified route is shown in Figure S1), yielding dark-red single-crystals that were successfully characterized via 1D ^1^H and ^13^C NMR, FT-IR, HRTOF-MS elemental analysis (see details in the Supplementary Materials, Figures S6–S12) and single-crystal X-ray diffraction (SC-XRD) (Figure 2).

The coordination bond parameters and three-dimensional Cartesian parameters are summarized in Table 1 and Table S1, respectively.

As we can see, by coordination with the metal, the salophen framework has undergone a considerable improvement in molecular planarity and symmetry, conferring molecular geometry that is close to the C_2v_ point group, as previously described elsewhere for Zn(II) coordination [51,53]. Nevertheless, in the case of the sal-3,4-ben ligand, regardless of the increase in planarity of the salophen moiety after coordination, its molecular structure still exhibits a non-symmetric system (C_1_ point group), due to the presence of the benzoyl moiety. However, considering the benzene ring of the benzophenone group, the C_2v_ point group symmetry should be noted (Figure 2). We also noted that a THF solvent molecule was inserted into the [Pt(sal-3,4-ben)] crystal structure during the crystallization process, changing its crystal group from orthorhombic (space group P2_1_2_1_2_1_) in the [Pt(salophen)] to monoclinic, with the space group P2_1_ (Figures S2 and S3).

The angles of the coordination site (Table 1), which are very close to 90°, have been observed for both compounds, whereas the O3-Pt1-N4/O2-Pt1-N5 angles are 179.009°/178.100° and 179.730°/177.730° for [Pt(salophen)] and [Pt(sal-3,4-ben)], respectively. These angle values confer a planar square molecular geometry on the Pt(II) cation coordination site that could create efficient recombination processes in terms of photo- and electroluminescence.

### 2.2. Optical and Theoretical Properties

The steady-state electronic absorption and photoluminescence (PL) spectra of [Pt(salophen)] and [Pt(sal-3,4-ben)] compounds in THF solutions (10 μmol L^−1^) are shown in Figure S5. Table S2 summarizes the respective data. The salophen and sal-3,4-ben free-ligand electronic spectra were detailed in previous works by [51,54], respectively. The electronic absorption spectra are composed of both the absorption bands of the ligands at higher energies and the bands of the Pt(II) at lower energies. Thus, whereas the electronic absorption spectra of the Zn(II) compounds are practically the same as those of the ligands [51,55], the spectral profile was significantly modified upon coordination with the Pt(II) cation. The lower-lying band is centered around λ_abs_ = 540–550 nm for both Pt(II) compounds. These differences could be related to the spin-orbit coupling, due to the presence of the heavy atom in the molecular structure, which completely modifies the ligands’ electronic structure.

**Figure 3:**
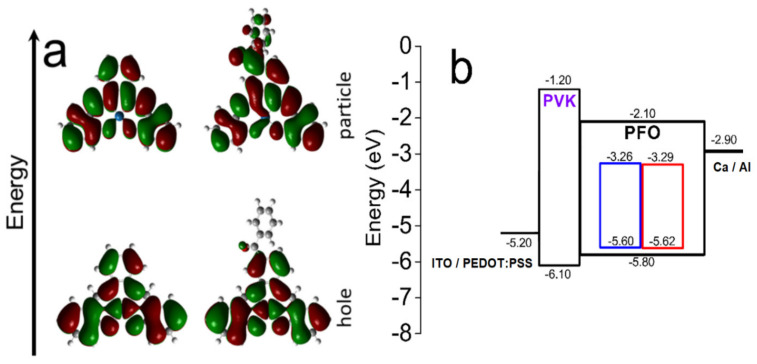
(**a**) NTOs hole/particle pair densities for the first triplet excited states of [Pt(salophen)] and [Pt(sal-3,4-ben)] in a vacuum at the PBE0/(def2-TZVP(C,N,O,H) and SARC-ZORA-def2-TZVP-Pt(II) atom) level (green and red colors represent the different density parities); (**b**) experimental OLED energy level diagram (inside the PFO energy diagram: blue represents [Pt(salophen)] and red represents [Pt(sal-3,4-ben)]).

The theoretical calculations used the molecular structure information of the Pt(II) coordination compounds obtained through SC-XDR. Thus, we obtained the vertical excitation energies (E/eV), oscillator strength (f), and molecular natural transition orbital (NTOs) densities for [Pt(salophen)] and [Pt(sal-3,4-ben)] (Figure 3a and Figure S4; the data are summarized in Table 2).

In the case of [Pt(salophen)], the energy calculation of the low-lying singlet electronic transition (S_0_ → S_1_) is 2.47 eV (501.3 nm; f = 0.079), assigning them only to the frontier molecular orbitals (HOMO → LUMO), was in agreement with the experimental data (2.37 eV; 524 nm). Further, the calculated energy of the first transition of [Pt(sal-3,4-ben)] was 2.39 eV (519.1 nm; f = 0.047), assigned to HOMO → LUMO electronic transition. This result presents a good correlation with the experimental value for the first electronic transition (2.31 eV; 536 nm). For both compounds, the NTOs hole/particle pair densities (Figure 3) showed a similar electronic density distribution in the ground-state (HOMO), having a minimal contribution from the benzophenone moiety. However, the presence of the benzophenone moiety considerably disrupts the electronic density of the particle (LUMO), and the unoccupied $5d_{x^{2}-y^{2}}$ orbital of Pt(II) to take part in the S_1_ density, which is delocalized, along with the π* framework. Therefore, the electronic transition of the [Pt(sal-3,4-ben)] exhibits an MLCT/ILCT mixed character.

The PL spectrum of the [Pt(salophen)] (λ_exc_ = 385 nm) in the THF solution (Figure S5a) has a band peaking at 621 nm, which is red-shifted in comparison to the free-salophen ligand [51]. There is also a substantial enhancement of the PLQY of the [Pt(salophen)] in THF saturated with N_2_(g), in contrast to the salophen-free ligand [51] (PLQY = 0.47). The calculated energy for the triplet state transition (T_1_ → S_0_; 1.97 eV; λ = 629 nm) matched well with the experimental data. The PL spectra of [Pt(sal-3,4-ben)] recorded in the THF solution (λ_exc_ = 389 nm; Figure S5b) has an emission peak at λ_em_ = 637 nm, with a 1.6 times higher phosphorescence quantum yield (PLQY = 0.76) in comparison to the [Pt(salophen)] under the same experimental conditions. In addition, the calculated energy transition from the T_1_ state (1.91 eV) shows an excellent correlation with the experimental data (1.93 eV).

The monoexponential phosphorescence decays of both compounds were also collected under the same experimental conditions (λ_exc_ = 375 nm) (Figures S13 and S14, Table S2), these being τ[Pt(salophen)] = 3.459 ± 0.001 μs, and τ[Pt(sal-3,4-ben)] = 2.747 ± 0.006 μs. Furthermore, the radiative and non-radiative constant rates (k_R_ and k_nR_, respectively) of the Pt(II) complexes were obtained by combining the photostationary and photodynamic rate equations [56] (Table S2). The k_R_ of the [Pt(sal-3,4-ben)] shows almost twice its analogous compound, while its non-radiative decay component exhibits one order of magnitude less than [Pt(salophen)]. This behavior is in accordance with the PLQY trend, indicating that [Pt(sal-3,4-ben)], with its high radiative rate, presents fewer non-radiative pathways, in contrast to the [Pt(salophen)] compound. This trend may be related to the presence of the benzoyl moiety, which, when linked to the salophen framework, confers a more efficient ISC process to it, increasing the triplet emission probability due to a better overlap between the S_1_ and T_1_ wavefunctions [57].

**Figure 4:**
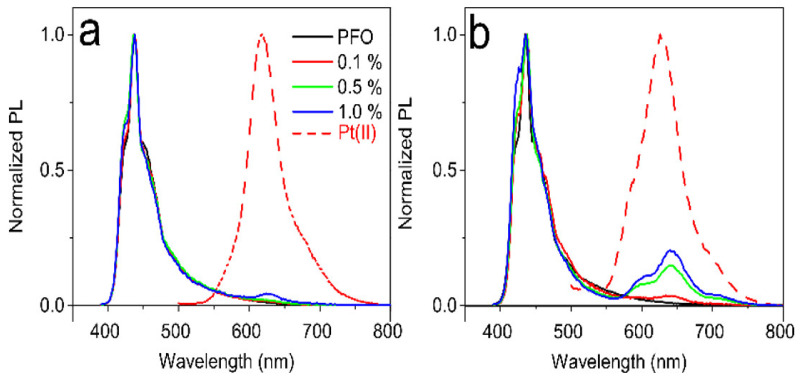
Normalized PL (λ_exc_ = 375 nm) spectra of the PFO composites. (**a**) [Pt(salophen)] and (**b**) [Pt(sal-3,4-ben)], with 0%, 0.1%, 0.5% and 1% mol/mol of complexes.

The PL spectra of both compounds were also studied in the composite PFO:[Pt(II)] under the same conditions as the OLED (host: guest) emissive layers (Figure 4). For both compounds, the PL spectra are very similar to those in the THF solutions, with emission bands centered at 628 nm and 643 nm for [Pt(salophen)] and [Pt(sal-3-4-ben)], respectively (Figure 3). The emission intensities are proportional to the amount of the compounds in the composites, which are 0.1%, 0.5%, or 1.0% mol/mol.

For comparison, we also recorded the PL spectrum of neat PFO (represented by the black curves in Figure 4). This spectrum is characteristic of PFO in a solid state, exhibiting a mixture of α-phase (amorphous) and β-phase, with the zero-phonon band at λ_amorphous_ = 425 nm and λ_β-phase_ = 437 nm, respectively [53,58,59]. The PFO spectra in the composites are different, indicating that the presence of the Pt(II) compounds impacts the PFO’s morphology. This change is more pronounced in the presence of [Pt(sal-3,4-ben)].

These optical studies lead us to the conclusion that both compound [Pt(salophen)] and compound [Pt(sal-3,4-ben)] are phosphorescent emitters at room temperature, which is a result of the T1 state and, according to the theoretical calculations, involves mixed ^3^MLCT/^3^ILCT/^3^MC character transitions. In composites with PFO, we observed both the blue emission from the PFO and the red emission from the Pt complexes, which cover almost the entire visible spectral region. Considering all these features, the composites of the PFO semiconductive polymer with phosphorescent Pt(salicylidenes) are excellent candidates for white light-generation OLEDs, as we will explore in the next section of the manuscript.

### 2.3. Solution-Processed Color Tunable WOLEDs

Solution-processed OLEDs require a great deal of effort to obtain a suitable host: guest structure, even more so if an active layer must be obtained using very low concentrations of the guest material. In our case, taking into account the photophysical properties of the Pt(salicylidenes) composites in the PFO semiconductive polymer, it would be expected that some simple WOLED architectures can be made with wet deposition. The main goal, besides an attempt to focus on our understanding of the common OLEDs’ solution-deposition physical processes, is to achieve a trade-off between practicality and usefulness and their main figures of merit. Typically, high efficiencies and bright OLEDs can easily be obtained in 4–6-layer devices, by means of common paper thermal evaporation [60]. Although yielding good results, the massive implementation that would be necessary, particularly in the case of lighting (i.e., large-area devices), appears to be complex and difficult. Thus, simplified structures, which are compatible with large-area and low-cost deposition methods (printing electronics), are, therefore, of great interest [61]. The structure and fabrication process of the WOLEDs presented in this work are shown in Figure S16 (Section S5) of the Supplementary Materials.

**Figure 5:**
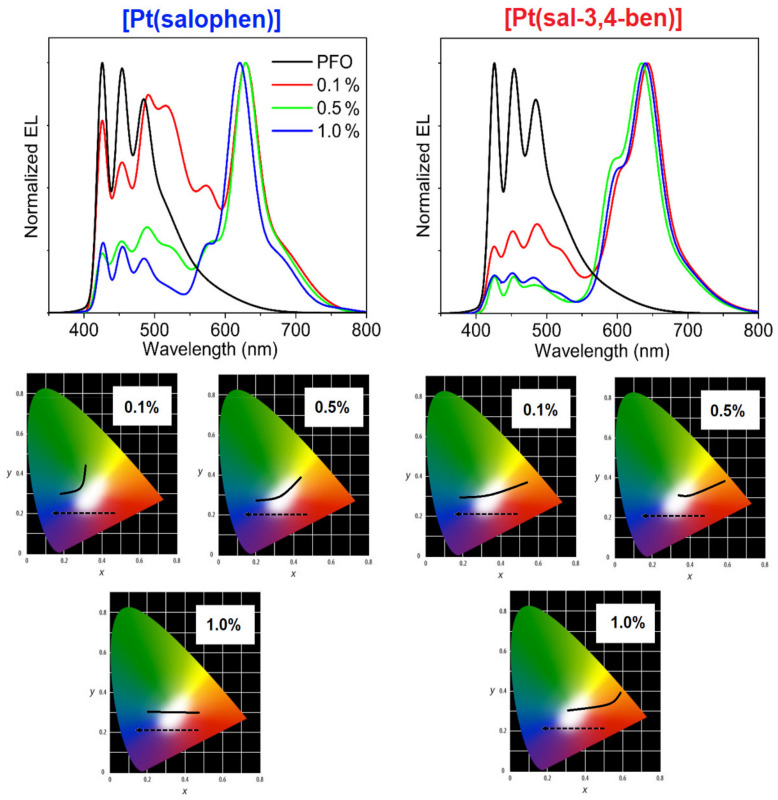
Electroluminescence spectra for [Pt(salophen)] and [Pt(sal-3,4-ben)] OLEDs with different concentrations, at maximum EQE. At the bottom, CIE1931 chromaticity coordinates the dependence on the bias of PFO:[Pt(II)]-based OLEDs for concentrations of 0.1, 0.5, and 1.0% of the Pt(II) complexes (arrows indicate the applied voltage increase and solid-lines represents the WOLEDs color change).

Figure 5 shows the EL spectra at the maximum EQE point and the CIE1931 (*x,y*) color coordinates, as a function of the applied voltage of all WOLEDs. It is clear that the EL and PL spectral profiles are remarkably different, particularly in the higher energy areas, where the emissions arise from PFO (see Figure 3). The PFO emission at λ_EL_ = 425 nm can be attributed to the zero-phonon band of isolated chains emitting in the PFO amorphous phase; it also has a well-known vibronic progression. In addition, a greenish band appears, λ_EL_ = 525 nm, which is due to the PFO chain aggregates [22]. This band is almost absent in the neat PFO [53]. These significant differences between the PFO emission profiles in the PL and EL spectra may be attributed to the different substrates used to process the emissive layer. PFO and its composite thin films were deposited on SiO_2_-coated glass substrates for the PL experiments, which can induce specific crystallization pathways in the PFO chains, as we can see in the presence of its β-phase emission. In contrast to the PL measurements, EL experiments were conducted within a much more complex architecture assembly, with three other layers before the emissive layer, the PFO, and its composites, deposited over the PVK layer. In addition, Quites et al. [62] observed that the PFO layer can interpenetrate into the PVK layer during the spin-coating process, which can dissolve well-organized PFO domains (crystalline β-phase) in the final electroluminescent device. An important finding is that the PFO aggregate emission is relatively more intense in the presence of [Pt(salophen)] compared to [Pt(sal-3,4-ben)], which suggests that these final compounds are intercalated between the polymer chains, inhibiting the π-π stacking. Thus, combining the amorphous PFO blue emission and its aggregate greenish emission (λ_EL_ ≈ 520 nm) with the red-phosphorescence of either [Pt(salophen)] (λ_EL_ = 629~620 nm) or [Pt(sal-3,4-ben)] (λ_EL_ = 646~636 nm) resulted in pure white light, as the chromaticity diagrams show in Figure 4, with CIE1931 (*x,y*) = (0.33, 0.33). This behavior is more evident with lower concentrations of the emitter (0.1% mol/mol).

As a general trend, the Pt(II) composites’ turn-on voltage is lower than that of the pure PFO device; the emitted color might be modulated by the bias, from the red color (at a lower bias) toward a blue-greenish color (at a higher bias), as presented in their chromaticity diagrams (Figure 5). Certain findings can be verified in the OLEDs EL spectra against the working voltages for all composite concentrations of [Pt(salophen)] (Figure S20) and [Pt(sal-3,4-ben)] (Figure S21): (1) the red emission band of Pt(sal-3,4-ben) dominates the EL spectra of its composites with PFO, even at very low concentrations (0.1%), while Pt(salophen) presents a true white EL color at the same concentration at very low working voltages; (2) the increment of the concentration of the Pt(II) compounds favors the red color in the devices in both cases, to the detriment of the PFO’s blue-greenish emissions, which necessitates a higher electrical field to achieve the same pure white color; (3) PFO’s blue-greenish EL emissions are predominant only at high voltages. These behaviors can be explained, not only by the fact that [Pt(sal-3,4-ben)] has a higher PLQY and offers better energy transfer from the PFO host than the [Pt(salophen)] compound but also by the energy level alignment of the active layer components. The energy levels of the active-matrix components were determined by cyclic voltammetry (Figures S17 and S18), the results being: E_HOMO_ = −5.60 eV and E_LUMO_ = 3.26 eV (E_gap_ = 2.34 eV), and E_HOMO_ = −5.62 eV and E_LUMO_ = −3.29 eV (E_gap_ = 2.33 eV) for [Pt(salophen)] and [Pt(sal-3,4-ben)], respectively (Figure 3 and  Figure S15). With these energy values, we can achieve a complete energy level diagram (Figure 3), including the frontier HOMO and LUMO of all other components of the OLEDs. According to these features, [Pt(salophen)] and [Pt(sal-3,4-ben)] turned on at a lower bias than PFO; this polymer starts emitting only when the charge-carriers in the valence and conduction bands of the coordination compounds become saturated. This is the reason why the diode turns bluish at a higher voltage.

**Figure 6:**
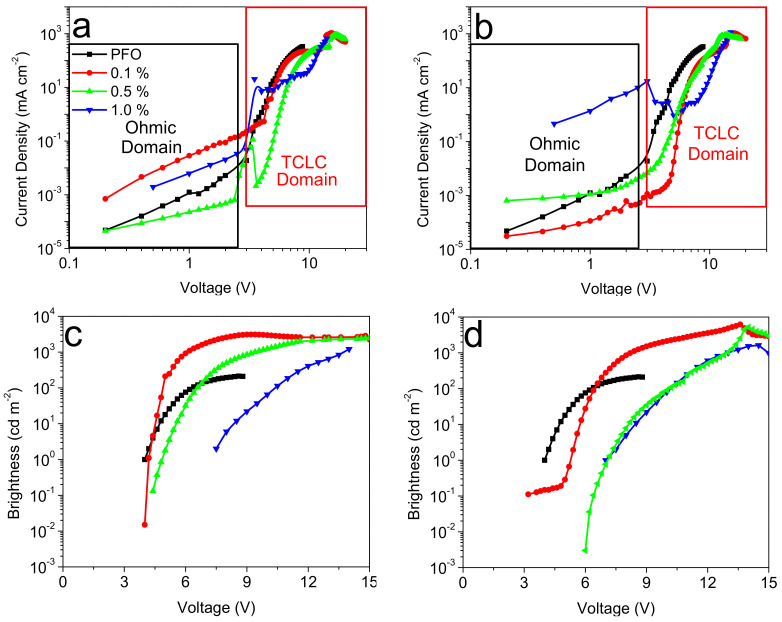
Optical-electronic properties of the diodes ITO|PEDOT:PSS/PVK/PFO:[Pt(salicylidene)s]/Ca|Al: current density and brightness vs. the voltage data of [Pt(salophen)] (**a**,**c**) and [Pt(sal-3,4-ben)] (**b**,**d**)-based OLEDs.

The current density-voltage-brightness (J-V-L) values of the [Pt(salophen)] (a and c) and [Pt(sal-3,4-ben)] (b and d) WOLEDs are shown in Figure 6. Their figures of merit and charge-carrier transport characteristics are summarized in Table 3.

The general behavior is almost the same for both guests. One typical influence of the active layer molecular conformation in the electrical carrier transport is clearly shown in the ohmic region (before the turn-on OLED voltage value) for some samples, where some I-V instability is present. This behavior is usual and disappears after several I–V cycles [63].

For devices that are based on the [Pt(salophen)] emitter, we can observe generally poorer optical-electronic characteristics; on the other hand, OLEDs based on [Pt(sal-3,4-ben)] achieve a maximum luminance of over 6224 cd m^−2^ (at 13.4 V) and with a lower turn-on voltage (V ≈ 3.2 V) (emitter concentration of 0.1% mol/mol). Overall, independently of the guest employed, the best performances are always obtained for that emitter concentration (which is particularly low). This behavior is particularly interesting as the usual OLEDs, based on efficient emitters (not only transition-metal complexes but also, for instance, thermally activated delayed fluorescence (TADFs)), usually have much higher guest concentrations (in some cases, up to 10–15 wt% before quenching—in our cases, the values are lower than 10^−3^–10^−2^ wt%). In order to attempt to explain these results, we need to understand the phase separation at the active layer, based on the chemical properties of the PFO polymer. Typically, such a blue-emitting polymer exhibits a particularly high molecular rigidity, in spite of using a fast evaporation solvent (THF), that would not be enough to avoid guest aggregates, with further losses of charge-carrier transport and radiative recombination (emission-quenching of the Pt(II) complexes) in the emissive layer. Nevertheless, for a so simple device structure, the figures of merit based on such a host polymer are interesting and can open the way to further frameworks. We will discuss this issue later in the paper.

From Figure 5, it is clear that when under an appropriate applied voltage, a white emission is obtained. Such behavior is to be expected when adding two complementary colors of emissions together, although, as is usual in such a situation, no stable white color in all applied voltages of the OLED working dynamic region is obtained. This is a direct consequence of using booth emitters with different recombination kinetics. Considering that, as shown for applied voltages over ≈4 V, almost the only emission is white, we can consider that in a suitable device operating voltage/brightness, we have created a WOLED. Figure 6 illustrates the OLEDs’ figures of merit in terms of the power efficiency, current efficiency, and EQE vs. brightness plots for each Pt(II) compound and their concentration inside the PFO host matrix. Effectively, and as a general trend, most diluted OLED active layers (0.1% mol/mol or ≈ 6 × 10^−4^ wt% of Pt(II) coordination compounds) offer greater performance than the 0.5 and 1.0%, as well as in the neat PFO device. Thus, the best figures of merit of the assembled OLEDs were obtained from PFO:[Pt(sal-3,4-ben)] as an active layer at 0.1% mol/mol, having a maximum current and power efficiencies of η_C_ = 12.1 cd A^−1^ and η_P_ = 11.9 lm W^−1^, respectively, and an EQE maximum of 15.4% (at 3.4 V), while the [Pt(salophen)]-based OLED at the same concentration shows poor performance parameters at the maxima efficiency points: V_on_ = 4.0 V; L_max_ = 3103 cd m^−2^ (9.2 V); η_C_ = 2.8 cd A^−1^; η_P_ = 1.8 lm W^−1^; and EQE = 2.2% (at 4.2 V). Notwithstanding the fact that such values seem to be relatively poor, the fact of the matter is that these values were obtained at a very low concentration of the emitter and in a very simplified device structure. Later, we will compare this performance with that of the state of the art.

**Figure 7:**
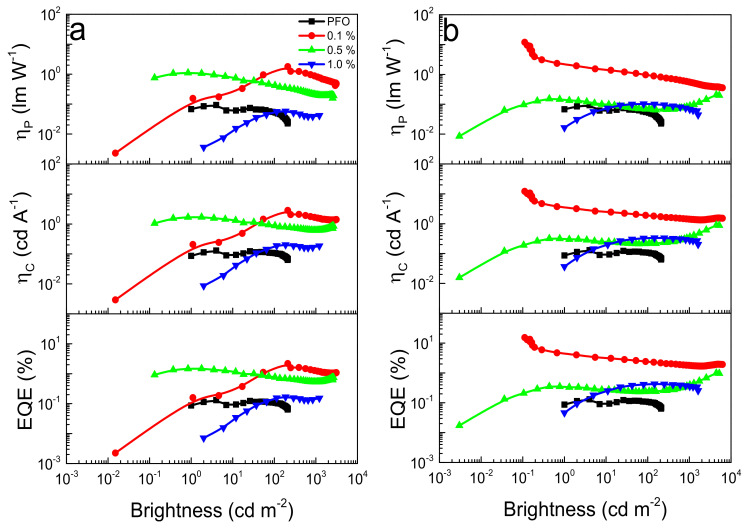
OLEDs figures of merit vs. brightness plots for PFO:[Pt(salophen)s] (**a**) and PFO:[Pt(sal-3,4-ben)] (**b**).

Although the PFO:[Pt(sal-3,4-ben)] (0.1%) exhibits high-efficiency opto-electronic properties in comparison with [Pt(salophen)] at the same concentration, a strong EQE roll-off (Figure S22) of about 80% is observed for 0.1% of [Pt(sal-3,4-ben)], along with the luminance rising: L_on_ @ 15%; L = 1 cd m^−2^ @ 3%; L = 10 cd m^−2^ @ 1.4%; L = 100 cd m^−2^ @ 1%; L = 1000 cd m^−2^ @ 0.6%; and L_max_ @ 0.4%. On the other hand, the WOLED based on 0.1% of [Pt(salophen)], although it presents low EQE and brightness values, has shown better roll-off (49%; Figure S22) than the [Pt(sal-3,4-ben)] diode: L_on_ = 1 cd m^−2^ @ 0.1%; L = 10 cd m^−2^ @ 1.2%; L = 100 cd m^−2^ @ 0.8%; L = 1000 cd m^−2^ @ 0.5%; and L_max_ @ 0.7%. Another important observation is that an OLED with a [Pt(sal-3,4-ben)] concentration of 0.5% has minimal working roll-off around its L_max_ point, once this device matches its maxima efficiencies parameter points with L_max_ (see Figure 7 and Table 3). All these figures of merit features can be explained by the photophysical, morphological and electric/electronic characteristics of the active layers, as we will discuss below.

As is well known, the optoelectrical OLEDs figures of merit are strongly dependent upon the three efficiencies: the emissive layer PLQY, electrical carrier balance, and opto-coupling effects. This work focuses on the first two efficiencies.

Regardless of the concentration, the luminance of the OLED with PFO:[Pt(sal-3,4-ben)] is twice that of the OLED with PFO:[Pt(salophen)]. More importantly, the efficiencies are beyond comparison due to the extreme ranges. One reason which might explain this greater performance is naturally related to the PLQY, which is twice that of [Pt(sal-3,4-ben)] compared to [Pt(salophen)]. As the EQE depends on the PLQY, and the architectures of the OLEDs are similar, it is expected that, regardless of other changes, the EQE of [Pt(sal-3,4-ben)]-based devices will be higher than that obtained by [Pt(salophen)]-based devices.

However, some other issues may also contribute to their different results, such as guest solubility (in the precursor solution and in the host matrix) and the morphological characteristics. The main impact of the organic layer’s morphology, particularly in terms of the active layer, is the usual and intrinsic defect creation, which, acting as a trap for the electrical carriers, unbalances the electrical transport equilibrium [64]. The primary consequence is the low EQE, although absolute values should be compared with those from a device with similar structural complexity. Naturally, in solution-processed OLEDs, which always use a simplified device structure, such issues appear to be much more relevant than the complex thermally evaporated OLEDs, where the electrical charge balance is conventionally obtained, matching the HOMO/LUMO levels with the addition of several other layers. In printing electronics, such an approach is not viable.

Optimizing the layer morphology in solution-deposited devices is a task based primarily on phase separation optimization, at which point the structural/electronic defects can be controlled. Therefore, knowledge of the electrically active defects (traps for electrical carriers) is of great interest to explain the obtained OLED figures of merit and gives feedback for further improvements. In particular, as the morphological aspects greatly impact the exciton recombination seen in the active layer (and corresponding device figures of merit), we will discuss that issue in terms of our OLEDs. Considering such questions, in this study, electron- and hole-only devices (Figure S19) were fabricated and electrically characterized to obtain true insights into the OLEDs’ active layers’ electrical properties, specifically in the 0.1% and 0.5% PFO:Pt(salicylidene) device characteristics (those for whom the figures of merit appear viable). In such a device, the electrical carriers’ transport (and trap dependence) properties can be estimated under space-charge limited-current (SCLC) conditions, according to the Mott–Gurney law given by [65]:

(1) $$J_{SCLC}=\frac{9}{8}\varepsilon_{r}\varepsilon_{0}\mu_{i^{SCLC}}\frac{V^{2}}{L^{3}}$$

where *J_SCLC_* is the current density in the SCLC region, *ε_r_* is the semiconductor layer relative dielectric constant (for almost of organic semiconductors *ε_r_* ≈ 3), *ε_0_* is the free-space permittivity, *μ_iSCLC_* is the electron (i = e) or hole (i = h) charge-carrier mobility in the SCLC region, *V* is the applied voltage, and *L* is the semiconducting layer’s thickness (~80 nm).

The SCLC model was applied in the trap-filling region of the device’s current density versus voltage curves (Figures S23 and S24), to obtain the electron and hole mobilities (*μ_eSCLC_* and *μ_hSCLC_*, respectively) during the trap-filling process. The PFO:[Pt(sal-3,4-ben)] active layer, with a concentration of 0.1% of the emitter, exhibits an enhancement of about 105 and 102 times that of the hole and electron mobilities, respectively, in comparison with the neat PFO device, this being *μ_hSCLC_* = 4.7 × 10^−5^ cm^2^ V^−1^ s^−1^ and *μ_eSCLC_* = 5.4 × 10^−10^ cm^2^ V^−1^ s^−1^, while the PFO:[Pt(salophen)], at the same concentration, did not present significant changes in both charge-carrier mobilities (*μ_hSCLC_* = 9.6 × 10^−10^ cm^2^ V^−1^ s^−1^ and *μ_eSCLC_* = 8.7 × 10^−12^ cm^2^ V^−1^ s^−1^). Hole mobility deteriorates in PFO:[Pt(salicylidenes)] from 0.1% to 0.5% of the Pt(II) complex inside the PFO matrix: *μ_hSCLC_* = 1.5 × 10^−9^ and 2.4 × 10^−10^ cm^2^ V^−1^ s^−1^ for [Pt(sal-3,4-ben)] and [Pt(salophen)], respectively. On the other hand, an increment of the Pt(salicylidenes) increases their electron mobilities by one order of magnitude (all the relevant data are summarized in Table 3, later). It becomes clear that our best OLEDs figures of merit with both emitters arise from the conjugation of the better relevant efficiencies: the photophysical characteristics and their charge-carrier mobilities from the space-charge model, although these are not absolutely addressed to just the one device type. The high PLQY of the [Pt(sal-3,4-ben)] guarantees its high L_max_ and efficiency values around the Von of their OLEDs, with 0.1% of the emitter; indeed, their great discrepancy in electron and hole mobilities during the trap-filling process leads to major roll-off behavior. On the other hand, due to [Pt(salophen)], OLEDs and more concentrated [Pt(sal-3,4-ben)] devices exhibit a better charge-carrier balance when their roll-offs are better, although presenting the worst figures of merit.

In order to obtain some insights regarding the charge-carrier mobility in the complete OLEDs, we applied a generalization of the Mott–Gurney model, which considers the trapped-charge limit transport on the device (trapped charge—limited current—TCLC) under space-charge conditions, as described by Equation (2) [66,67]:

(2) $$J_{TCLC}=q^{1-l}\mu^{TCLC}N_{F}{\left(\frac{2l+1}{l+1}\right)}^{l+1}{\left(\frac{l}{l+1}\frac{\varepsilon \varepsilon_{0}}{N_{t}}\right)}^{l}\frac{V^{l+1}}{L^{2l+1}}$$

where *q* is the elementary electrical charge, *μ^TCLC^* is the electrical mobility, *N_F_* is the density of free carriers, *N_t_* is the total density of the trap-states, and *l* is the trap energy parameter (*l* = E_t_/kT; E_t_ is the trap energy, k is the Boltzmann constant, and T is the temperature). *N_t_* values can be obtained from the trap-filling limit voltage (*V_TFL_*) via a simple estimation of the defects (traps) densities, which is given by:

(3) $$V_{TFL}=\frac{qL^{2}N_{t}}{2\varepsilon_{r}\varepsilon_{0}}$$

**Figure 8:**
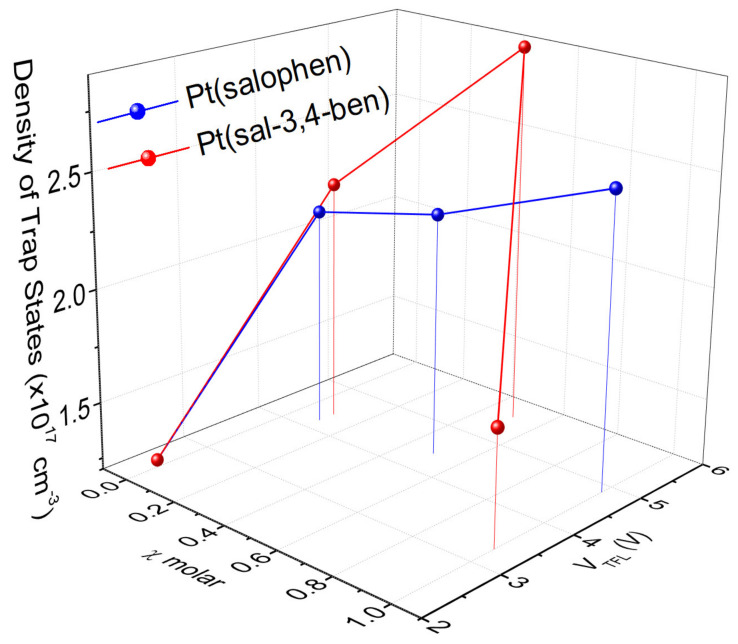
Density of the trap states, as determined by the VTFL data for both OLEDs’ emissive layers.

Figure 8 shows the estimated trap densities as a function of *V_TFL_*, along with the concentration of both Pt complexes in the emissive layer. It is noteworthy to verify that although the trap densities are relatively high (in the order of 10^17^ cm^−3^) for both coordination compounds, the fast increase in the molar concentration of [Pt(salophen)] is noticeable; on the other hand, for small concentrations (0.1% mol/mol), there are no significant changes, indicating that the poor OLEDs figures of merit obtained with such a guest have arisen from what is clearly the worst host: guest phase separation.

From the described models, we can estimate the total electrical charge mobility for the OLED with PFO:[Pt(sal-3,4-ben)] as μ(0.1) = 2.8 × 10^−3^ cm^2^ V^−1^ s^−1^, which is significantly higher than that for PFO (μ_PFO_ = 5.3 × 10^−4^ cm^2^ V^−1^ s^−1^) which can, in conjunction with the differences in PLQY, explain the greater performance of the [Pt(sal-3,4-ben)]-based OLED. The values of the mobilities follow the trend for the OLED efficiencies of PFO:[Pt(sal-3,4-ben)] and decreases with increasing [Pt(sal-3,4-ben)] concentration: μ(0.1%) = 2.8 × 10^−3^ > μ(0.5%) = 5.7 × 10^−4^ > μ(1.0%) = 2.8 × 10^−6^ cm^2^ V^−1^ s^−1^. Furthermore, the carriers’ mobilities are lower for the OLED of PFO:[Pt(salophen)] compared to PFO:[Pt(sal-3,4-ben)] and the pure PFO devices, in agreement with performance. These results show, naturally, that devices based on [Pt(sal-3,4-ben)] exhibit the rapid drift transport of charge-carriers in the trap-filling processes on the full OLED, although showing poor electron: hole balance behavior, which, when conjugated with their photophysical features, may explain the overall figures of merit.

An insight into the nature of trap-states on the PFO:[Pt(salicylidene)] OLEDs can be obtained by estimating the values of the trap energy under SCLC and TCLC conditions, as explained before. As a general trend, the addition of the Pt(II) coordination compounds increases the trap energy of the active layer, exhibiting values that are about hundreds of meV, which is typical in the presence of deep-trap levels. Nevertheless, it is expected that the device’s active layers exhibit charge transfer processes from the polymer host to the Pt(II) complex guest. As the emitter energy levels can act as deep-trap levels during the charge transport in the active matrix, the presence of these deep-trap levels may not affect the OLEDs’ performance, once the Pt(II)salicylidenes can perform triplet harvesting. Table 3 summarizes the optoelectrical properties of the OLEDs.

Two interesting key points need to be highlighted. First, the OLEDs almost exhibit a low roll-off (see Figure 5) with the best device (with 0.1% mol/mol of [Pt(sal-3,4-ben)]); this is not usual in simple OLED structures that are based on transition metal complexes, due to the long lifetime of the triplet excited state. In this case, one of the main contributors to the roll-off is the triplet-triplet annihilation (TTA) [68], which can explain some of the roll-off behavior in our case (see Figure S22a,b), although the lifetimes are in agreement with the usual ones found in transition-metals emitters that are used in efficient devices; moreover, the triplet levels of both Pt complexes (3.4 eV for Pt(salophen) and 3.5 eV for Pt(sal-3,4-ben)) are far from the PFO triplet level (≈2.8 eV), avoiding resonance. The second important issue is the clear relationship between the emissive layer’s molecular conformation and the obtained results. As highlighted, there is a systematic decrease in the device performance at higher concentrations, which can be attributed (not the only issue, but surely one of the most important ones) due to changes in the film morphology produced by the aggregation of the coordination compounds. Not only does the formation of the aggregates lead to the occurrence of a bad phase separation with both [Pt(salophen)] and [Pt(sal-3,4-ben)] complexes but the overall electrical charge mobility also degrades with the expected increase in intrinsic defects that act as traps, contributing to the poorer performance (a decrease in the electrical carrier balance efficiency) with the concentration increment and high roll-off of the 0.1% of [Pt(sal-3,4-ben)].

As is well known, the difference in trap density between both kinds of active layers is one of the most important reasons for seeing so huge a difference in the figures of merit in the corresponding OLEDs. Several factors directly impact the final device efficiency, namely, the trap density (from the intrinsic defects), dopant concentration and molecular structure, and exciton confinement in the active layer, as well as how the host: guest phase separation is achieved. In this work, it is evident that each of these aspects does not act independently; on the contrary, they are dependent, although some of the factors can be more dominant. In the first approximations, and considering the ideal model for the host: guest matrix, it can be supposed that the dopant acts as a trap site for the electrical carriers. Unfortunately, in some situations, the molecular conformation (and local symmetry) in the host: guest molecular domain leads to a trapping charge, but with non-radiative recombination. This type of behavior seems to be the situation when [Pt(salophen)] is the guest. On the other hand, we can see deep-trap states in our devices where we can expect to have localized electronic states that are not thermally activated. This implies that their existence leads to the modification of the local electrostatic equilibrium, which will impact directly upon the exciton recombination and on the final device’s figures of merit. In our OLEDs, the HOMO/LUMO differences between the guests and the host are relatively similar for both complexes; moreover, the T_1_ states are far enough apart to avoid resonance. Considering the fact that the guest concentration is low enough to minimize self-quenching and the excited lifetime is short enough for phosphorescence OLEDs to avoid triplet-triplet annihilation (although in the case of [Pt(salophen)], such a lifetime is longer, opening up some possibilities for TTA annihilation), the results seem to be trap-related; in turn, they may be intrinsically dependent on the molecular structure of the guests. Moreover, the PLQY, as indicated, is clearly lower in [Pt(salophen)]; thus, we expect worse device performance.

**Figure 9:**
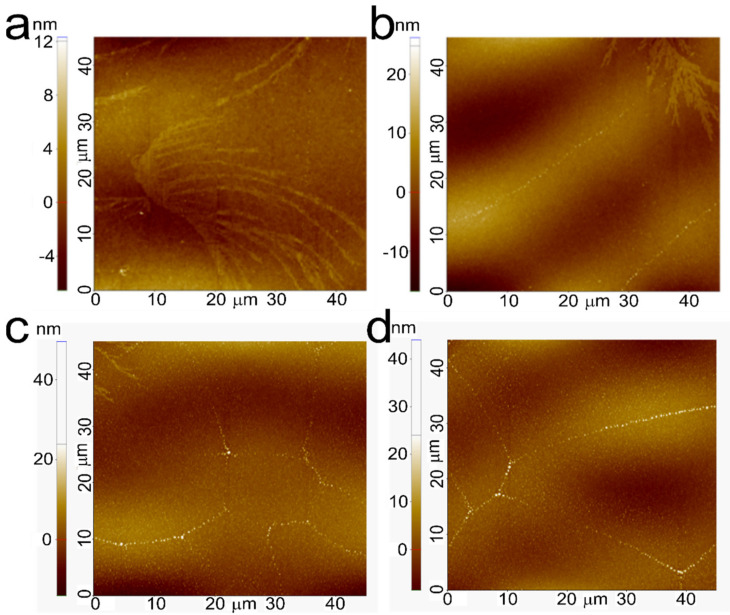
AFM topography images (45 × 45 μm^2^) of the PFO:Pt(salicylidenes) emissive layers, on top of the ITO|PEDOT:PSS|PVK layers: [Pt(salophen)] (**a**,**b**) and [Pt(sal-3,4-ben)] (**c**,**d**) at 0.1% and 0.5%, respectively.

PFO is a polymer with a very rigid molecular structure and with a strong crystallization phase. Naturally, the presence of relatively large crystalline domains can help in electrical charge transport and are the reason that PFO was chosen (other blue-emitter polymers traditionally exhibit very low electrical charge mobility). On the other hand, this high rigidity is an important issue when optimizing the OLEDs. As pointed out earlier, even with fast solvent evaporation, avoiding bad phase separation due to the tendency of the PFO to crystallize in large and rigid domains when doped with the Pt(II) complexes, we expected to see that non-perfect phase separation can occur with a further decrease in the OLED figures of merit, as the AFM topography images reveal in Figure 9. In this context, the [Pt(salophen)] structure has the worst solubility in the PFO host matrix, providing a bad topological morphology. Cumulatively with low PLQY, this should not be the ideal complex for the simplified structure of an OLED, where the PFO hosts the active layer. On the other hand, [Pt(sal-3,4-ben) exhibits much better solubility interaction with the PFO host, which agrees with its greater device charge-carrier mobilities and global figures of merit.

Finally, some observations regarding the overall efficiencies versus device viability should be made. Our OLEDs have only two organic layers (regardless of PEDOT:PSS, a standard “metal-like” polymer that was merely used to optimize the anode, just as Ca was employed to optimize the cathode) with PVK used as an electron-hole-blocking layer. The highest EQE obtained (15.3% for the [Pt(sal-3,4-ben)] emitter) near V_on_, although decreasing by nearly 2.5% for a luminance of around 0.1 cd/m^2^, remains almost constant up to nearly 1000 cd m^−2^, which is one of the best roll-offs known for so simple a device structure based on Pt complexes. The control of the solution-processed organic layers (in terms of host: guest materials, concentration, solvent, and deposition parameters), shows that a decrease in the trap densities, with more efficient electrical carrier balance due to the better molecular conformation of the active layer, may be the main explanation. A simple comparison of the figures of merit of the devices that were created with other previously published works involving Pt-based emitters (Table S3), which are mainly produced by the thermal evaporation of the layers and contain auxiliary layers to boost performance, can be achieved. It can be observed that our devices represent an advance due to being constituted by a single emissive layer, with minimal quantities of the guest material, in a truly simple device structure. With the above-mentioned tuning of the host: guest properties impacting the molecular morphology conformation, it is possible to achieve performances that correspond to a very good trade-off between device simplicity and efficiency. Moreover, by taking advantage of the host’s emissions, it is also possible to tune the final emitting color, as with the white emission (WOLED) shown in this work.

## 3. Conclusions

In this work, we synthesized and characterized Pt(II) coordination compounds with salicylidene-derivative ligands. These compounds were dissolved in PFO at different concentrations to form the OLEDs’ active layers, which were assembled using the all-solution process method. These coordination compounds exhibited a planar square coordination geometry that will directly impact their optical properties, due to the higher planarity and rigidity of the molecular framework compared with the free ligand. The theoretical calculation showed that the first singlet-state transition (HOMO → LUMO) is composed mainly of ILCT/MLCT and ILCT/MLCT/MC for [Pt(salophen)] and [Pt(sal-3,4-ben)], respectively, with the contribution of the filled 5*d_x-y_* Pt(II) orbital on the ground state for the two coordination compounds and the unoccupied $5d_{x^{2}-y^{2}}$ orbital. The optimal concentration of Pt(II) compounds in the PFO matrix (host: guest configuration) is 0.1% mol/mol (≈6 × 10^−4^ wt%) for the performance of those OLEDs, with a simplified structure of ITO|PEDOT:PSS/PVK/PFO:Pt(II) Ca|Al. The OLED performance of the PFO:[Pt(sal-3,4-ben)] is higher than that of the OLEDs with PFO:[Pt(salophen)] at the same concentration, which can be explained by their greater PL quantum yield, charge mobilities, and molecular conformation in a phase separation with reduced trap density. White emissions were successfully generated by the combination of EL emissions from both the PFO and Pt(II) coordination compounds since they have complementary colors, with a minimal roll-off of the OLEDs’ principal figures of merit, mainly for the PFO:[Pt(sal-3,4-ben)] active matrix at low concentrations. With these results, a good trade-off between simplicity and efficiency was achieved with a simple device architecture/easy processability of the active matrix and created efficient, useful devices. Understanding the physics of the charge transport and transfer between the PFO and the phosphorescent coordination compounds, with the corresponding feedback for the device fabrication process and the related parameters, appears to be the principal matter to be elucidated. Further developments based on such a framework can be performed at a later date.

## Figures and Tables

**Table 1:** Chemical bond distances to the coordination site, from the single-crystal X-ray data of the [Pt(salophen)] and [Pt(sal-3,4-ben)] parameters.

Bonds	[Pt(salophen)]	[Pt(sal-3,4-ben)]
**Pt1–O2**	1.983(4) Å	1.984(2) Å
**Pt1–O3**	1.991(4) Å	1.994(2) Å
**Pt1–N4**	1.965(5) Å	1.959(2) Å
**Pt1–N5**	1.955(4) Å	1.953(2) Å
**N–Pt1–N**	83.830°	83.727°
**O–Pt1–O**	85.258°	86.380°
**N5–Pt1–O3**	95.509°	95.437°
**N4–Pt1–O2**	95.587°	95.440°
**O3–Pt1–N4**	179.003°	179.730°
**O2–Pt1–N5**	178.100°	177.730°

**Table 2:** Theoretical energy excitations of [Pt(salophen)] and [Pt(sal-3,4-ben)], calculated in the DFT/TD-DFT framework at the PBE0/(def2-TZVP(C,N,O,H) and SARC-ZORA-def2-TZVP-Pt(II) atom) level in a vacuum (* larger contributions, ** first triplet state).

[Pt(salophen)]				[Pt(sal-3,4-ben)]			
E/eV	λ/nm	*f*	Assignment *	E/eV	λ/nm	*f*	Assignment *
2.47	501.3	0.079	H → L	2.39	519.1	0.047	H → L
3.02	410.7	0.045	H−1 → L	2.81	441.0	0.034	H → L + 1
3.60	344.5	0.750	H−2 → L	2.95	420.7	0.019	H−1 → L
3.69	336.5	0.250	H−1 → L + 1	3.46	358.9	0.650	H−2 → L
4.08	303.6	0.079	H−2 → L + 1	3.55	349.3	0.180	H → L + 2
1.97 **	629.4			3.64	341.0	0.001	H−6 → L
				1.91 **	647.7		

**Table 3:** PFO:[Pt(salicylidenes)] WOLED charge-carrier characteristics and the figures of merit. (^a^ Values of the λ_EL_ at the maximum peak band of each EL spectrum).

	%	λ_EL_ (nm) ^a^	V_on_	μ_hSCLC_ (cm^2^ V^−1^ s^−1^)	μ_eSCLC_ (cm^2^ V^−1^ s^−1^)	N_t_ (cm^−3^)	μ^TCLC^ (cm^2^ V^−1^ s^−1^)	E_t_ (meV)	L_max_ (cd m^−2^)	η_curr_ (cd A^−1^)	η_p_ (lm W^−1^)	EQE_max_ (%)
PFO		425	4.0	3.7 × 10^−10^	3.3 × 10^−11^	1.23 × 10^17^	5.3 × 10^−4^	226.2	213	0.1	0.1	0.1
[Pt(salophen)]	0.1	629	4.0	9.6 × 10^−10^	8.7 × 10^−12^	2.17 × 10^17^	8.5 × 10^−5^	175.0	3103	2.8	1.8	2.2
	0.5	629	4.4	2.4 × 10^−10^	5.8 × 10^−11^	2.28 × 10^17^	1.4 × 10^−5^	262.8	2670	1.7	1.1	1.5
	1.0	620	7.5	-	-	2.51 × 10^17^	6.5 × 10^−7^	191.4	1214	0.2	0.6	0.2
[Pt(sal-3,4-ben)]	0.1	643	3.2	4.7 × 10^−5^	5.4 × 10^−10^	2.27 × 10^17^	2.8 × 10^−3^	241.5	6224	12.1	11.9	15.3
	0.5	639	5.8	1.5 × 10^−9^	7.4 × 10^−10^	2.90 × 10^17^	5.7 × 10^−4^	260.8	5227	0.9	0.2	1.0
	1.0	636	7.0	-	-	1.71 × 10^17^	2.8 × 10^−6^	236.1	1622	0.3	0.1	0.4

## Data Availability

Not applicable.

## Supplementary Materials

The following supporting information can be downloaded at: https://www.mdpi.com/article/10.3390/nano12142497/s1, All materials and experimental and theoretical details are found in the Supplementary Materials. Figure S1. Scheme of the reactions in [Pt(salicylidene)], [Pt(salophen)] and ([Pt(sal-3,4-ben)]) syntheses; Figure S2. Crystal structure in the plane (**a**), out of the plane (**b**), and the cell packing of the [Pt(salophen)] (**c**) ellipsoids, with 50% probability; Figure S3. Crystal structure in the plane (**a**), out of the plane (**b**), and the cell packing of the [Pt(sal-3,4-ben)] (**c**) with THF solvent ellipsoids, with 50% probability; Figure S4. Front side visualization of the Pt(II) 5d-orbitals and ligand π-orbitals on the NTO-hole and particle densities; Figure S5. Normalized electronic absorption and phosphorescence spectra of: (**a**) [Pt(salophen)] (λ_exc_ = 385 nm), and (**b**) [Pt(sal-3,4-ben)] (λ_exc_ = 389 nm) in the THF solutions (10 μmol L^−1^); Figure S6. Electronic absorption spectra of [Pt(salophen)] in THF solutions as a function of the concentration: 1.10 × 10^−5^ (pink curve), 8.86 × 10^−6^ (blue curve), 6.68 × 10^−6^ (green curve), 4.47 × 10^−6^ (red curve) and 2.25 × 10^−6^ mol L^−1^ (black curve) (**a**); and molar absorption coefficient determination, via the Lambert–Beer law (**b**); Figure S7. Electronic absorption spectra of [Pt(sal-3,4-ben)] in THF solutions as a function of the concentration: 3.98 × 10^−6^ (pink curve), 3.20 × 10^−6^ (blue curve), 2.41 × 10^−6^ (green curve), 1.61 × 10^−6^ (red curve) and 8.11 × 10^−7^ mol L^−1^ (black curve) (**a**); and molar absorption coefficient determination via the Lambert–Beer law (**b**); Figure S8. The ^1^H NMR spectrum of the [Pt(salophen)] solution in DMSO-d6 (33 mg mL^−1^); Figure S9. The ^13^C NMR spectrum of [Pt(salophen)] solution in DMSO-d6 (33 mg mL^−1^); Figure S10. FT-IR spectra of [Pt(salophen)] and [Pt(sal-3,4-ben)] in ATR mode; Figure S11. TOFHR-MS spectra of [Pt(salophen)] in positive mode; Figure S12. The ^1^H NMR spectrum of the [Pt(sal-3,4-ben)] solution in DMSO-d6 (33 mg mL^−1^); Figure S13. The ^13^C NMR spectrum of the [Pt(sal-3,4-ben)] solution in DMSO-d6 (33 mg mL^−1^); Figure S14. The TOFHR-MS spectra of [Pt(sal-3,4-ben)] in the positive mode; Figure S15. Phosphorescence decays of [Pt(salophen)] (**a**) λ_exc_ = 375 nm; (**b**) λ_PL_ = 621 nm in the THF solution (10 μmol L^−1^) under N_2_(g)- and air-saturated conditions; Figure S16. Phosphorescence decays of [Pt(sal-3,4-ben)] (**a**) λ_exc_ = 375 nm; (**b**) λ_PL_ = 637 nm in the THF solution (10 μmol L^−1^) under N_2_(g)- and air-saturated conditions; Figure S17. Cyclic voltammograms of [Pt(salophen)] and [Pt(sal-3,4-ben)], measured in ACN so-lution (1 × 10^−4^ mol L^−1^); Figure S18. The Fc/Fc^+^ internal standard cyclic voltammogram, measured in ACN solution, and its half-wave potential (E_1/2_ = 0.5645 V); Figure S19. PFO:Pt(II)salicylidenes solution-processed WOLEDs, *p*- and *n*-type devices; Figure S20. Dependence of the ITO|PEDOT:PSS|PVK|PFO:Pt(salophen) 0.1, 0.5, and 1.0% (mol/mol)|Ca|Al WOLEDs electroluminescence spectra, with the applied bias; Figure S21. Dependence of the ITO|PEDOT:PSS|PVK|PFO:Pt(sal-3,4-ben) 0.1, 0.5 and 1.0% (mol/mol)|Ca|Al WOLEDs electroluminescence spectra, with the applied bias; Figure S22. EQE roll-off during working brightness in PFO-based WOLEDs assembled with 0.1 (black curves) and 0.5% (reference curves) of [Pt(salophen)] (**a**) and [Pt(sal-3,4-ben)] (**b**); Figure S23. Current density vs. the voltage curves of hole- (black squares) and electron-only (red circles) devices for 0.1% and 0.5% of the [Pt(sal-3,4-ben)]-based WOLEDs. Straight lines represent the best curve simulation, according to the Mott–Gurney SCLC model; Figure S24. Current density vs. the voltage curves of hole- (black squares) and electron-only (red circles) devices for 0.1% and 0.5% of the Pt(salophen)-based WOLEDs. Straight lines represent the best curve simulation, according to the Mott–Gurney SCLC model; Table S1. Main [Pt(salophen)] and [Pt(sal-3,4-ben)] Cartesian coordinates (*x,y,z*) of the O-Pt-N atom coordination site, according to the experimental single-crystal X-ray refinement; Table S2. Summary of the optical properties of [Pt(salophen)] and [Pt(sal-3,4-ben)] coordination compounds in THF solutions; Table S3. The optical-electronic parameters of OLEDs with Pt(II) complexes as a guest, where V_on_ is the turn-on voltage (V), L is the maximum luminance (cd m^−2^), J is the current density (mA cm^−2^), η is the current efficiency (cd A^−1^), and EQE (%). EML stands for the emissive layer. References [69,70,71,72,73,74,75,76,77,78,79,80,81,82,83,84,85,86,87] are cited in the Supplementary Materials.
