# Supplementary material for: All-Solution Processed Single-Layer WOLEDs Using [Pt(salicylidenes)] as Guests in a PFO Matrix
Source: Nanomaterials (Basel). 2022 Jul 20;12(14):2497. doi: 10.3390/nano12142497 (PMC9316125; doi:10.3390/nano12142497)
Supplement: Supplementary file 1 [file nanomaterials-12-02497-s001.zip › nanomaterials-1797615-supplementary.pdf]

nanomaterials

Supplementary Data

All-Solution Processed Single-layer WOLEDs Using [Pt(salicylidenes)] as Guests in a PFO Matrix

José Carlos Germino [1,2,*], Luís Gustavo Teixeira Alves Duarte [1], Rodrigo Araújo Mendes [3], Marcelo Meira Faleiros [1], Andreia de Morais [4], Jilian Nei de Freitas [4], Luiz Pereira [2,*] and Teresa Dib Zambon Atvars [1]

1 Chemistry Institute, University of Campinas - UNICAMP, Campinas 13083-862, Brazil; lg.alvesduarte@gmail.com (L.G.T.A.D.); mmfaleiros@gmail.com (M.M.F.); tatvars@unicamp.br (T.D.Z.A.)
2 Department of Physics and i3N - Institute for Nanostructures, Nanomodelling and Nanofabrication, University of Aveiro, 3810-193 Aveiro, Portugal
3 São Carlos Institute of Chemistry, University of São Paulo - USP, São Carlos 13566-590, Brazil; rodrigoaramendes@gmail.com
4 Center for Information Technology Renato Archer - CTI, Campinas 13069-901 Brazil; andreiademoraes6@gmail.com (A.d.M.); jilian.freitas@cti.gov.br (J.N.d.F.)
* Correspondence: germino@ua.pt (J.C.G.); luiz@ua.pt (L.P.)

This Supplementary Data contains all experimental and computational details: ^{1}H and ^{13}C NMR and FTIR spectra, TOFHR-MS spectrometry, electronic absorption, Lambert–Beer plot, photoluminescence spectra (PL), emission decay, and the cyclic voltammogram of the [Pt(II)] coordination compounds.

1. Materials

PVK (M_W = 1,100,000 g mol^{-1}), potassium tetrachloroplatinate(II) (K_2PtCl_4), tris(bipyridine)ruthenium(II) chloride ($[Ru(bipy)_3]Cl_2$), dimethylformamide (DMF), DMSO-d_6 and THF (anhydrous inhibitor-free grade) were purchased from Sigma-Aldrich, São Paulo, Brazil. Dimethyl sulfoxide (DMSO) was supplied by Tédia, Rio de Janeiro, Brazil, at HPLC/spectrum grade. Sodium acetate trihydrate was purchased from Synth, Diadema, Brazil. PFO (ADS129BE; M_w = 40,000~150,000 g mol^{-1}) was supplied by the American Dye Source Inc, Baie-D'Urfe, QU, Canada. PEDOT:PSS (Clevios™ P VP Al 4083) was purchased from Heraeus Group, Hanau, Germany. Salophen was synthesized and characterized elsewhere [1,2].

2. Synthesis of [Pt(salicylidenes)]

The [Pt(salicylidene)] synthesis was adapted from the study by Zhou et al. [3] (Figure S1). In a 25-mL three-neck round-bottomed flask, a dimethylformamide (DMF) (15 mL) solution of salicylidene ligand (0.5 mmol) and sodium acetate trihydrate (1 mmol; 82 mg) were stirred under an N_2(g) atmosphere at 70 ºC for 20 min. A DMSO (3 mL) solution of K_2PtCl_4 (0.5 mmol; 207 mg) was added dropwise and the reactional mixture was stirred for 12h. Upon cooling, deionized water (50 mL) was added to the mixture, then the red precipitate was filtrated, dried, and purified by recrystallization in the DMF:THF solvent mixture, yielding dark red crystals.

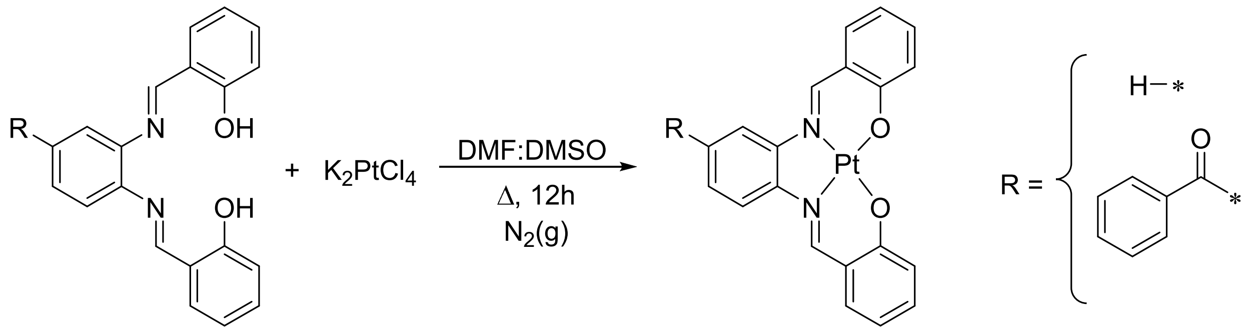

Figure S1. Scheme of the reactions in [Pt(salicylidene)], [Pt(salophen)] and ([Pt(sal-3,4-ben)]) syntheses.

[Pt(salophen)]: yield 219 mg, 86%. ^{1}H NMR (500 MHz, DMSO-d$_6$) δ (ppm): 9.53 (s, 2H), 8.46 (dd, J = 6.2, 3.4 Hz, 2H), 7.88 (d, J = 8.0 Hz, 2H), 7.58 (t, J = 7.7 Hz, 2H), 7.46 (dd, J = 6.3, 3.1 Hz, 2H), 7.13 (d, J = 8.6 Hz, 2H), 6.80 (t, J = 7.3 Hz, 2H) (Figure S1); ^{13}C NMR (126 MHz, DMSO-d$_6$) δ (ppm): 165.0, 151.8, 145.1, 136.2, 136.0, 128.5, 122.4, 121.7, 117.3, 116.8 (Figure S2). The main infrared bands in the ATR mode were (cm^{-1}): $\nu_{Pt\text{-}N}$ = 466, $\nu_{Pt\text{-}O}$ = 543, $\nu_{C=N}$ = 1601, $\nu_{C\text{-}O}$ = 1327, $\nu_{(C\text{-}H)Ar}$ = 3012–3086, and ν_{Ar} = 844 and 749 (Figure S3). HRTOF-MS ES+ (*m*/*z*): 510.0717 (Figure S4) was calculated for [M-H]$^+$: 510.0776. Elemental analysis was calculated (%) for $C_{20}H_{14}N_2O_2Pt$: C 47.15, H 2.77, N 5.50; found (%): C 47.16, H 2.86, N 5.44.

[Pt(sal-3,4-ben)]: yield 257 mg, 84%. ^{1}H NMR (500 MHz, DMSO-d$_6$): 9.55 (s, 1H), 9.54 (s, 1H), 8.56 (d, J = 8.7 Hz, 1H), 7.88 (m, 5H), 7.72 (m, 2H), 7.60 (m, 5H), 7.16 (t, J = 8.5 Hz, 2H), 6.80 (dt, J = 17.8, 6.9 Hz, 2H) (Figure S5); ^{13}C NMR (126 MHz, DMSO-d$_6$) δ 195.0, 165.5, 165.2, 152.9, 152.8, 147.8, 145.1, 137.2, 136.7, 136.5, 136.4, 136.4, 136.2, 133.7, 130.5, 129.5, 129.3, 122.5, 122.4, 122.0, 121.7, 118.1, 117.1, 117.1, 116.9 (Figure S6). The principal infrared bands in the ATR mode were (cm^{-1}): $\nu_{Pt\text{-}N}$ = 460, $\nu_{Pt\text{-}O}$ = 555, ν_{Ar} = 751 and 824, $\nu_{C=N}$ = 1603 and 1592, $\nu_{C=O}$ = 1661, $\nu_{C\text{-}O}$ = 1329, and $\nu_{(C\text{-}H)Ar}$ = 3031 – 3049 (Figure S3). TOF-MS (*m*/*z*): 614.0939 (Figure S7) was calculated for [M-H]$^+$: 614.1038. Elemental analysis calculated (%) for $C_{27}H_{18}N_2O_3Pt$: C 52.86, H 2.96, N 4.57; found (%): C 52.75, H 3.09, N 4.53.

3. Methods

The ^{1}H and ^{13}C NMR spectra of [Pt(salophen)] and [Pt(sal-3,4-ben)] in DMSO-d$_6$ solution (33 mg mL^{-1}) were measured using a Bruker Avance 500 MHz spectrometer.

The FTIR spectra were recorded using an Agilent Technologies Cary 630 spectrometer in attenuated total reflectance (ATR) mode. Elemental analysis (%) of the C, H, and N atoms was performed using a Perkin–Elmer microanalyzer, model PE 2400.

High-resolution time-of-flight mass spectrometry was acquired via a Xevo G2 QTof spectrometer with an *m*/*z* work range of 460–572, coupled with a WATERS ultra-performance liquid chromatograph (UPLC). HRTOF-MS was obtained from an acetonitrile solution (1% of formic acid), with a mist flow of 100 L h^{-1}, using the ES+ mode.

The crystal structures of [Pt(salophen)] and [Pt(sal-3,4-ben)] were determined by single-crystal X-ray diffraction in a Bruker Apex Duo X-ray diffractometer, using Mo (Kα = 0.71073 Å) radiation, with software for structural and unit cell refinements: APEX2 [4], SAINT [4], SHELXS97 [5], SHELXL2014/7 [6], and Mercury [7].

The absorption spectra of the [Pt(salicylidene)] coordination compounds, both in solution and in PFO film, were acquired with a Hewlett-Packard 8452A diode array spectrophotometer. Steady-state photoluminescence (PL) spectra were recorded with a Cary Eclipse Varian spectrofluorimeter using λ_{exc} = 375 nm to excite the PFO in the PFO:*X* film composites, and the λ_{exc} = 385 nm and λ_{exc} = 390 nm to excite [Pt(salophen)] and [Pt(sal-3,4-ben)] in the solutions, respectively. Samples of the films were oriented in a back-facing configuration at 45º. Spectra of the solutions were recorded using a 1-

centimeter quartz cuvette. Relative PL quantum yields ($PLQY_{obs}$) of both [Pt(salophen)] and [Pt(sal-3,4-ben)] in solutions were obtained using the relative method, according to Equation 1, using a tris(bipyridine)ruthenium(II) chloride ($[Ru(bipy)_3]Cl_2$) coordination compound (λ_{exc} = 450 nm; $PLQY_S$ = 0.094) diluted to the same concentration in acetonitrile as is standard, according to the IUPAC standards [8]:

$$PLQY_{obs} = \frac{F_i}{F_s}\frac{\varepsilon_s}{\varepsilon_i}\frac{n_i^2}{n_s^2}PLQY_s \quad (1)$$

where F_i and F_S are the integrated emission spectra of the sample and of the standard, ε_i and ε_s are their absorbances at the excitation wavelength of the measurement, and n_i and n_s are the refractive indexes of the solvents with which the compounds were diluted.

Phosphorescence decay of the [Pt(salicylidenes)] in the solutions (THF and EPA) was recorded using time-correlated single-photon counting (TCSPC) in an Edinburgh Analytical Instruments FL 900 spectrofluorometer, using an MCP-PMT (Hamamatsu R3809U-50) with a pulsed diode laser operating at λ_{exc} = 375 nm (model EPL-370, with a bandwidth of 5 nm, 77 ps). The decay signals were collected at λ_{PL} = 613, 621, and 625 nm, and at λ_{PL} = 626, 637, and 641 nm for [Pt(salophen)] and [Pt(sal-3,4-ben)], respectively. The instrument response was recorded using Ludox samples. At least 1000 counts in the peak channel were accumulated for the purposes of lifetime determination. The emission decays were analyzed using a single exponential decay function.

The redox potentials of the Pt(II) compounds were determined by cyclic voltammetry, using a model PAR 273A potentiostat with platinum working and counter electrodes and an Ag/AgCl reference electrode, in an acetonitrile/tetrabutylammonium hexafluorophosphate solution (0.1 mol L^{-1}). The scan rate for the cyclic voltammograms was 20 mV s^{-1}. The formal standard potential of the Fc^+/Fc redox couple in acetonitrile was approximately −5.1 eV. The HOMO (E_{HOMO}) and LUMO (E_{LUMO}) energies were also determined using Equations 2 and 3 [9].

$$E_{HOMO} = -\left(E_{\left[onset,ox\ vs.\frac{Fc+}{Fc}\right]} + 5.1\right) \quad (2)$$

$$E_{LUMO} = -\left(E_{\left[onset,red\ vs.\frac{Fc+}{Fc}\right]} + 5.1\right) \quad (3)$$

The atomic force microscopy (AFM) topography images were performed using a PARK XE7 atomic force microscope from Park Systems, coupled with a standard contact-mode AFM probe.

4. Theoretical Approach

Theoretical calculations of the electronic properties of both [Pt(salophen)] and [Pt(sal-3,4-ben)] were performed under the DFT/TD-DFT framework, using density-functional PBE0 [10], with the relativistic effects being within the zeroth-order regular approximation (ZORA). These approaches were employed using a relativistic all-electron Gaussian basis def2-TZVP to O, N, C, and H atoms [11], and segmented all-electron relativistic-contracted (SARC) Gaussian-type basis sets were assigned to Pt [12]. [Pt(salicylidenes)] X-ray single-crystal structures were used for the initial structure. Molecular structures corresponding to the ground states (S_0) were optimized in the gas phase. Singlet vertical electronic excitations and the first triplet natural transition orbitals (NTOs) pair were also obtained in the gas phase. All calculations were performed using the ORCA 4.0.1 open academic software, Frank Neese Group, Max Plank Institute, Mülheim an der Ruhr, Germany [13].

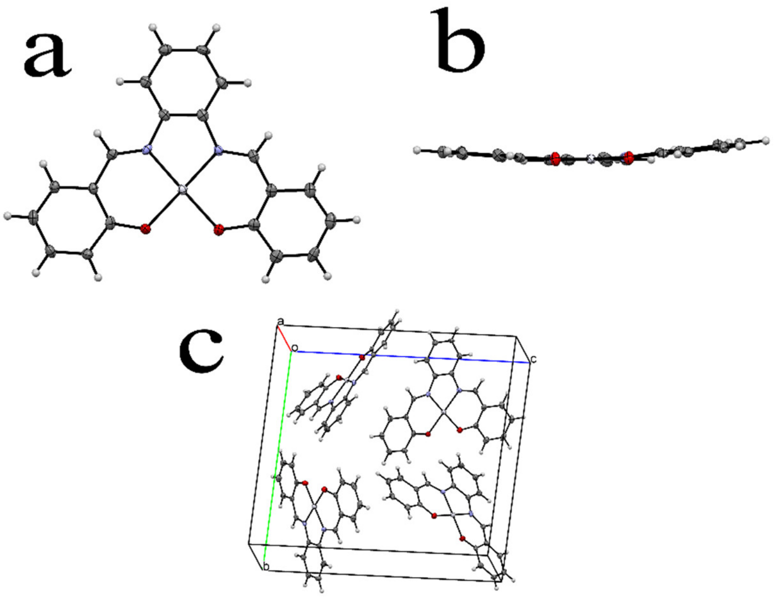

Figure S2. Crystal structure in the plane (**a**), out of the plane (**b**), and the cell packing of the [Pt(salophen)] (**c**) ellipsoids, with 50% probability.

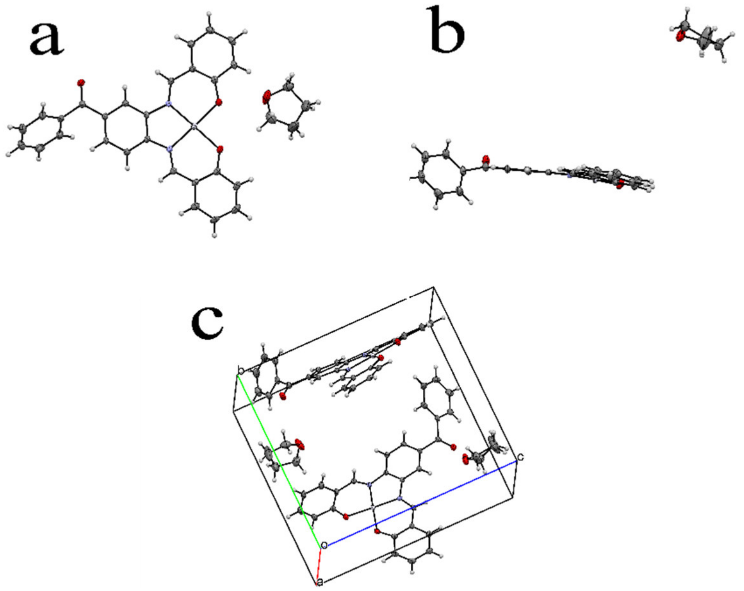

Figure S3. Crystal structure in the plane (**a**), out of the plane (**b**), and the cell packing of the [Pt(sal-3,4-ben)] (**c**) with THF solvent ellipsoids, with 50% probability.

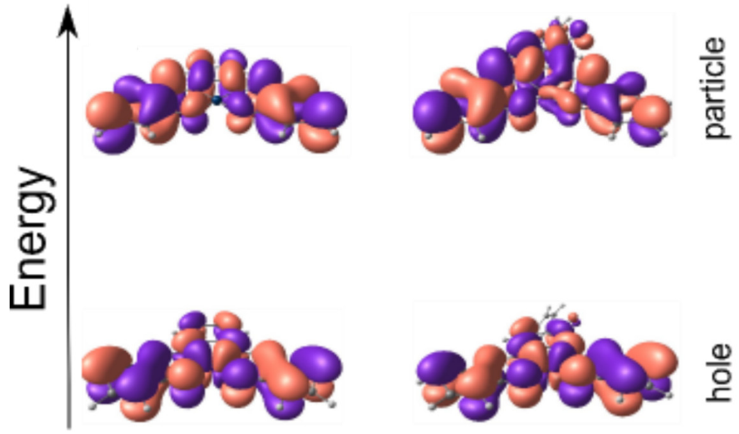

Figure S4. Front side visualization of the Pt(II) 5d-orbitals and ligand π-orbitals on the NTO-hole and particle densities.

Table S1. Main [Pt(salophen)] and [Pt(sal-3,4-ben)] Cartesian coordinates (*x*,*y*,*z*) of the O-Pt-N atom coordination site, according to the experimental single-crystal X-ray refinement.

[Pt(salophen)]			
**Atom**	**x**	**y**	**z**
Pt1	0.34259 (4)	0.28306 (2)	0.69175 (2)
O2	0.3515 (8)	0.3876 (2)	0.6395 (2)
O3	0.0437 (8)	0.3266 (2)	0.7469 (2)
N4	0.6391 (9)	0.2390 (3)	0.6389 (2)
N5	0.3447 (10)	0.1809 (3)	0.7449 (2)
**[Pt(sal-3,4-ben)]**			
Pt1	−0.15330 (2)	0.77547 (2)	0.59169 (2)
O2	−0.1503 (4)	0.7888 (2)	0.72457 (15)
O3	−0.4382 (4)	0.70176 (15)	0.59538 (16)
N4	−0.1513 (4)	0.7629 (3)	0.46139 (16)
N5	0.1332 (5)	0.84343 (17)	0.58692 (18)

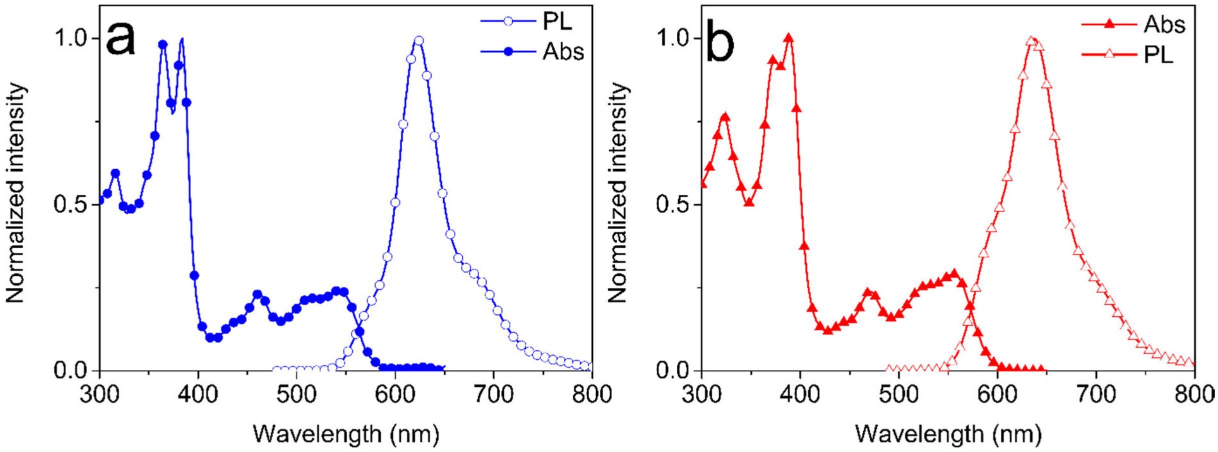

Figure S5. Normalized electronic absorption and phosphorescence spectra of: (**a**) [Pt(salophen)] (λ_{exc} = 385 nm), and (**b**) [Pt(sal-3,4-ben)] (λ_{exc} = 389 nm) in the THF solutions (10 μmol L^{-1}).

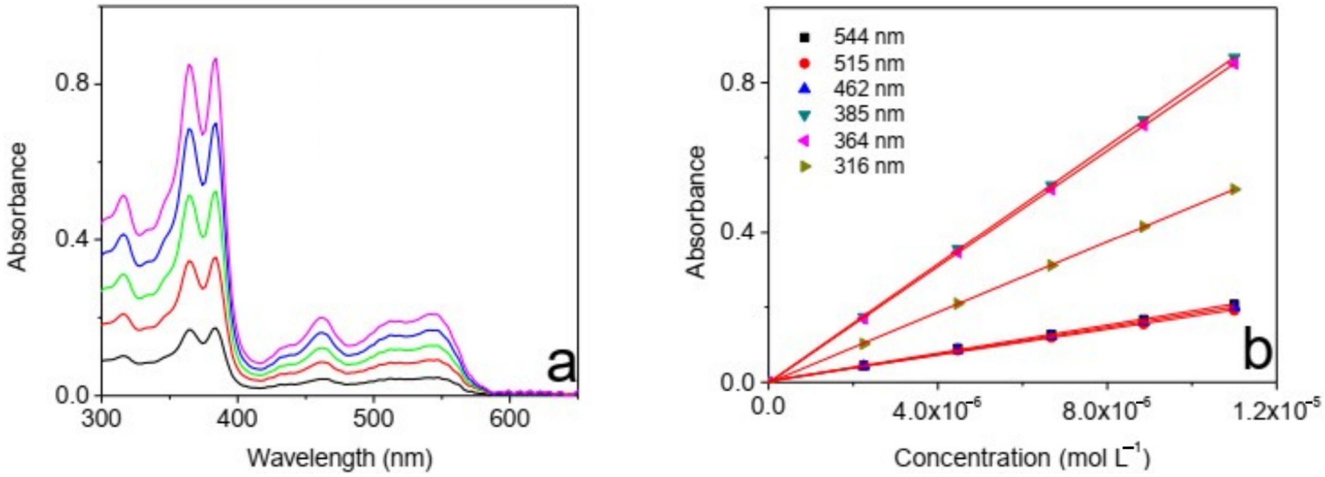

Figure S6. Electronic absorption spectra of [Pt(salophen)] in THF solutions as a function of the concentration: 1.10×10^{-5} (pink curve), 8.86×10^{-6} (blue curve), 6.68×10^{-6} (green curve), 4.47×10^{-6} (red curve) and 2.25×10^{-6} mol L^{-1} (black curve) (**a**); and molar absorption coefficient determination, via the Lambert–Beer law (**b**).

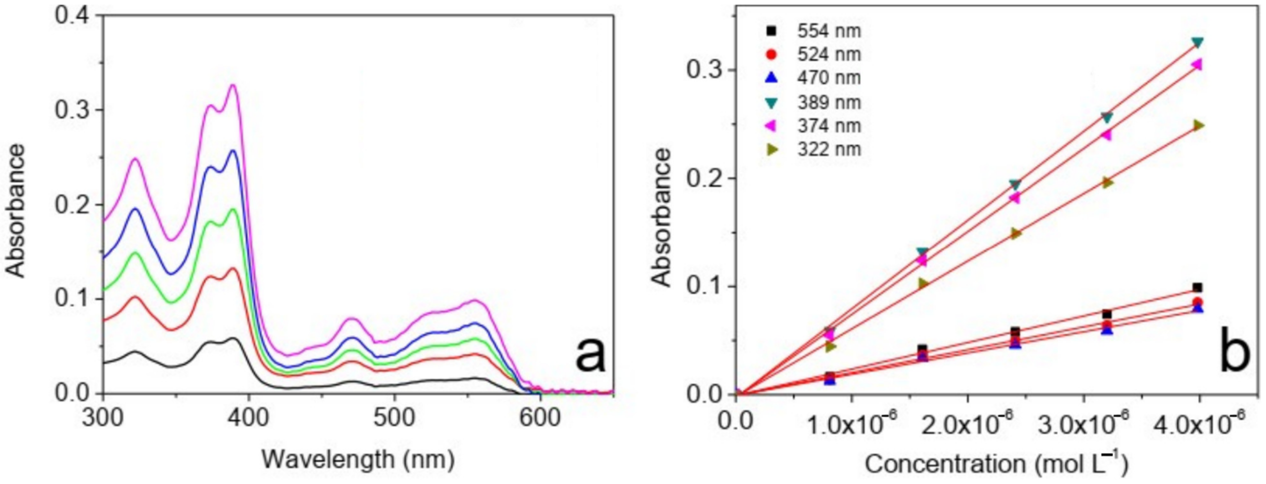

Figure S7. Electronic absorption spectra of [Pt(sal-3,4-ben)] in THF solutions as a function of the concentration: 3.98×10^{-6} (pink curve), 3.20×10^{-6} (blue curve), 2.41×10^{-6} (green curve), 1.61×10^{-6} (red curve) and 8.11×10^{-7} mol L^{-1} (black curve) (**a**); and molar absorption coefficient determination via the Lambert–Beer law (**b**).

Table S2. Summary of the optical properties of [Pt(salophen)] and [Pt(sal-3,4-ben)] coordination compounds in THF solutions.

.

λ_{abs1}/nm (ε/L mol^{-1} cm^{-1})	λ_{abs2}/nm (ε/L mol^{-1} cm^{-1})	λ_{abs3}/nm (ε/L mol^{-1} cm^{-1})	λ_{abs4}/nm (ε/L mol^{-1} cm^{-1})	λ_{abs5}/nm (ε/L mol^{-1} cm^{-1})	λ_{abs6}/nm (ε/L mol^{-1} cm^{-1})	λ_{em}/ nm	Φ_{phos}	τ_{phos}/μs	k_R/s^{-1}	k_{nR}/s^{-1}	*SS*/cm^{-1}
**[Pt(salophen)]**											
544 (1.87×10^4)	515 (1.72×10^4)	462 (1.81×10^4)	385 (7.90×10^4)	364 (7.75×10^4)	316 (4.70×10^4)	621	0.47	3.459±0.001	1.36×10^5	1.53×10^5	2279
**[Pt(sal-3,4-ben)]**											
554 (2.45×10^4)	524 (2.11×10^4)	470 (1.96×10^4)	389 (8.21×10^4)	374 (7.68×10^4)	322 (6.26×10^4)	637	0.76	2.747±0.006	2.77×10^5	8.74×10^4	2351

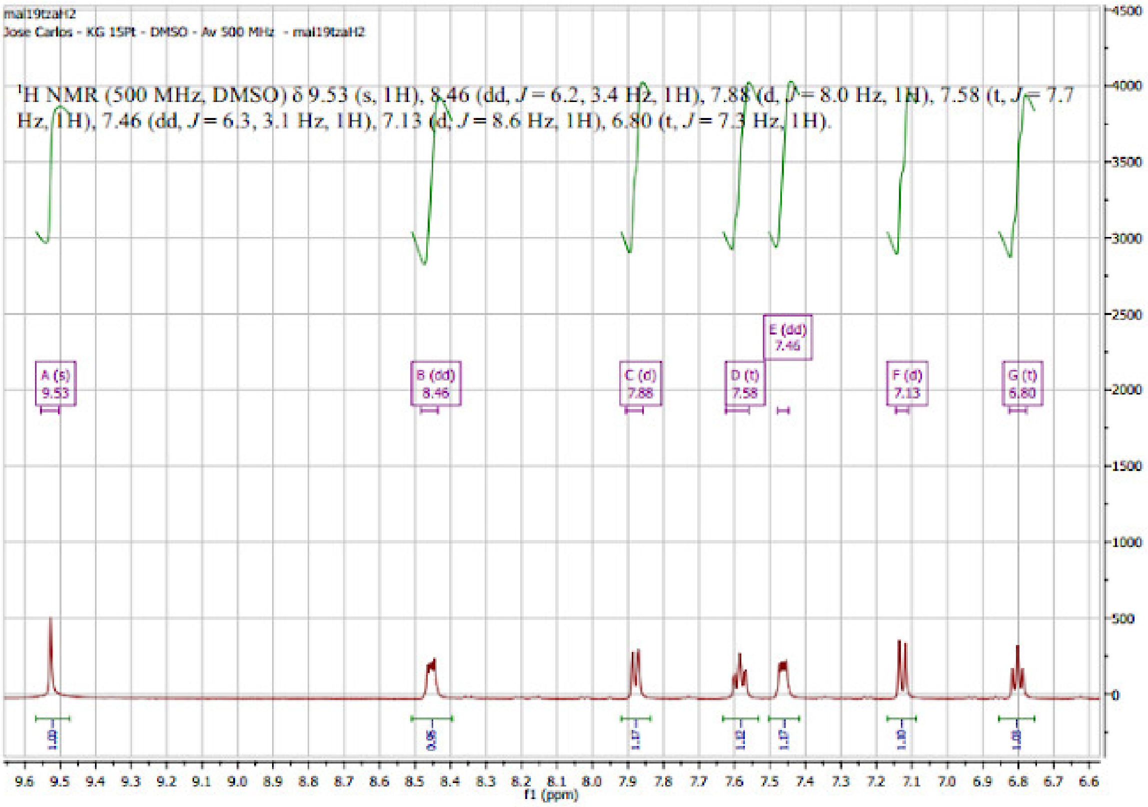

.

Figure S8. The ^{1}H NMR spectrum of the [Pt(salophen)] solution in DMSO-d_6 (33 mg mL^{-1}).

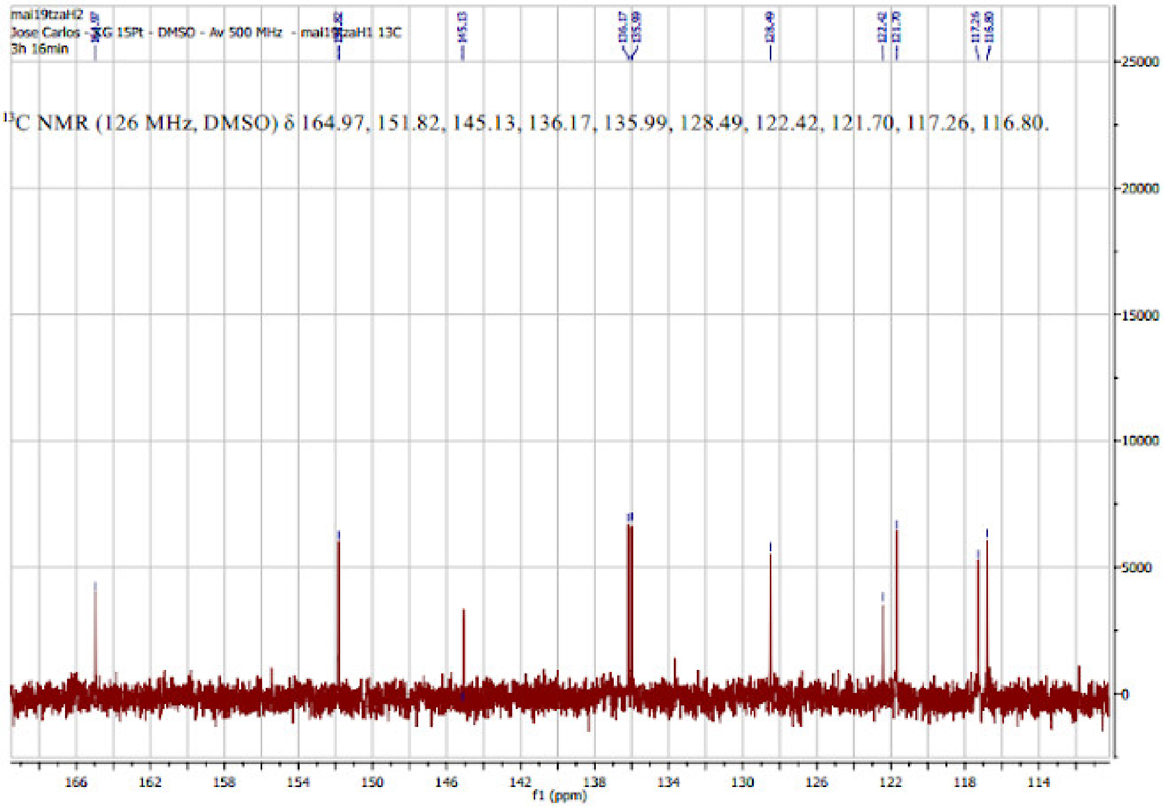

Figure S9. The ^{13}C NMR spectrum of [Pt(salophen)] solution in DMSO-d_6 (33 mg mL^{-1}).

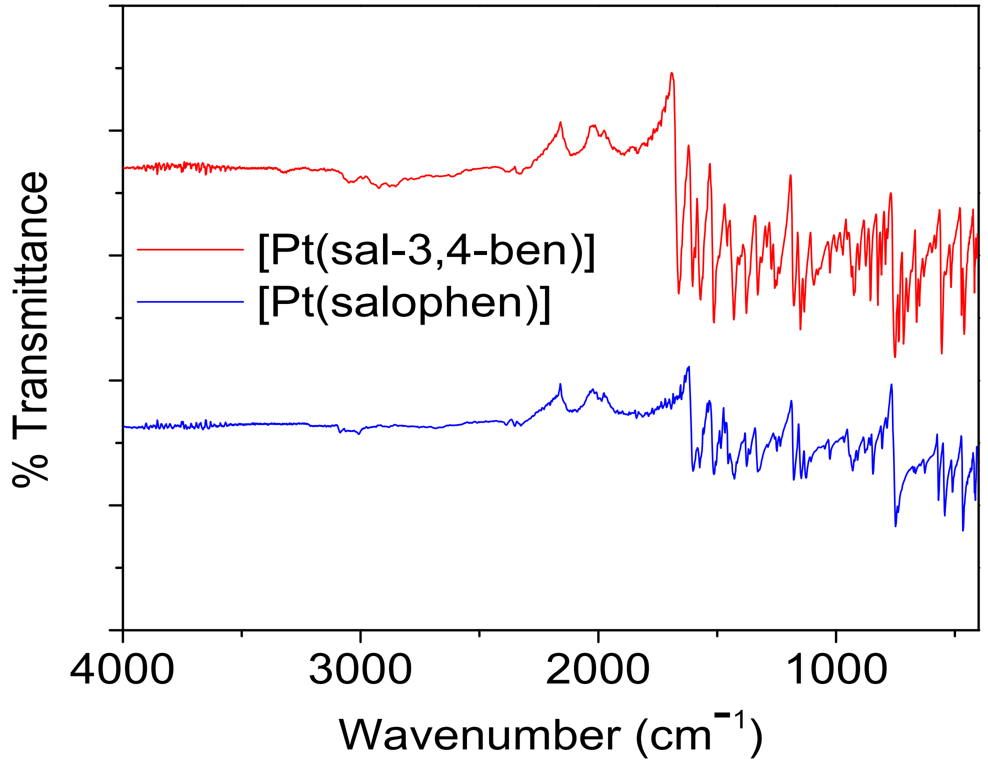

Figure S10. FT-IR spectra of [Pt(salophen)] and [Pt(sal-3,4-ben)] in ATR mode.

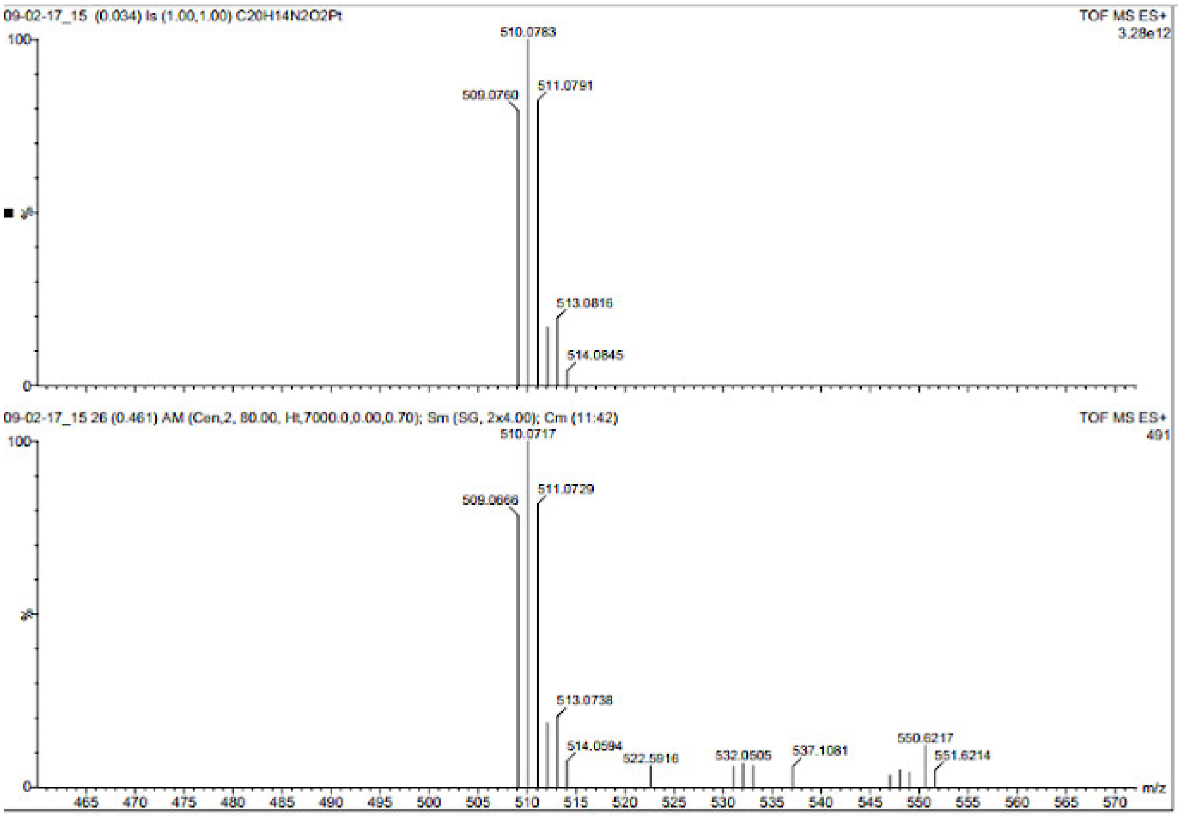

Figure S11. TOFHR-MS spectra of [Pt(salophen)] in positive mode.

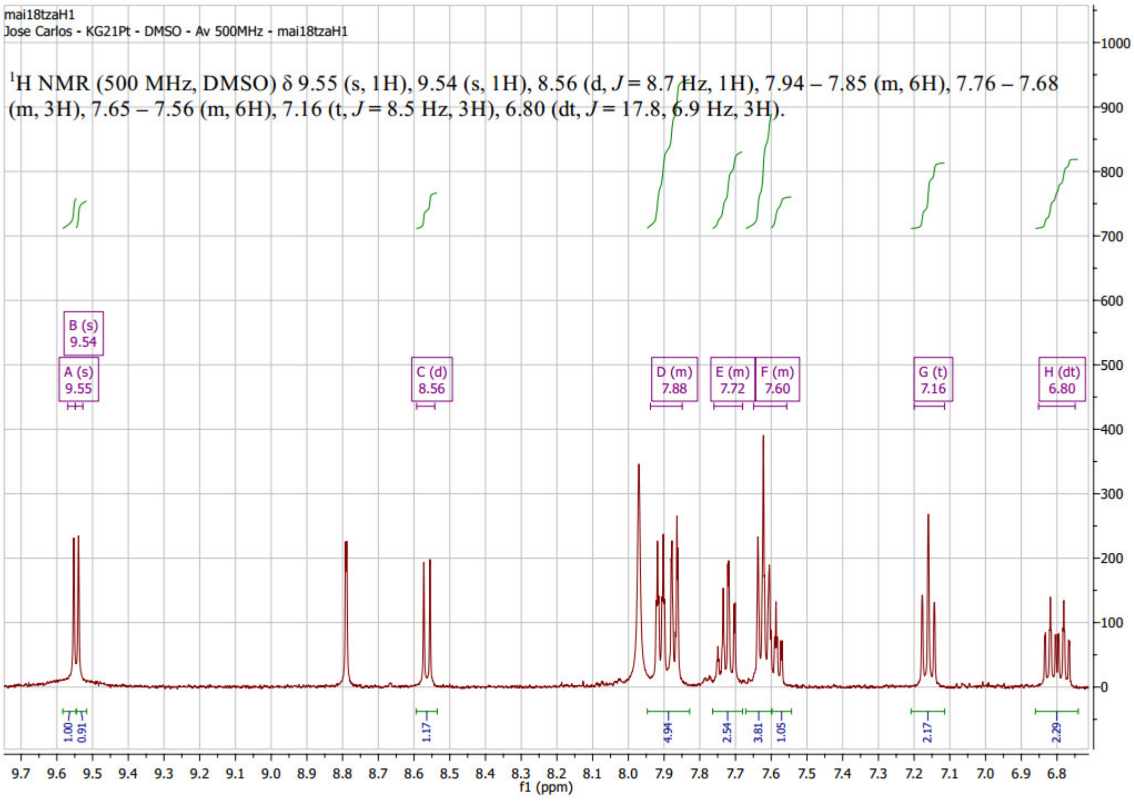

Figure S12. The ^{1}H NMR spectrum of the [Pt(sal-3,4-ben)] solution in DMSO-d_6 (33 mg mL^{-1}).

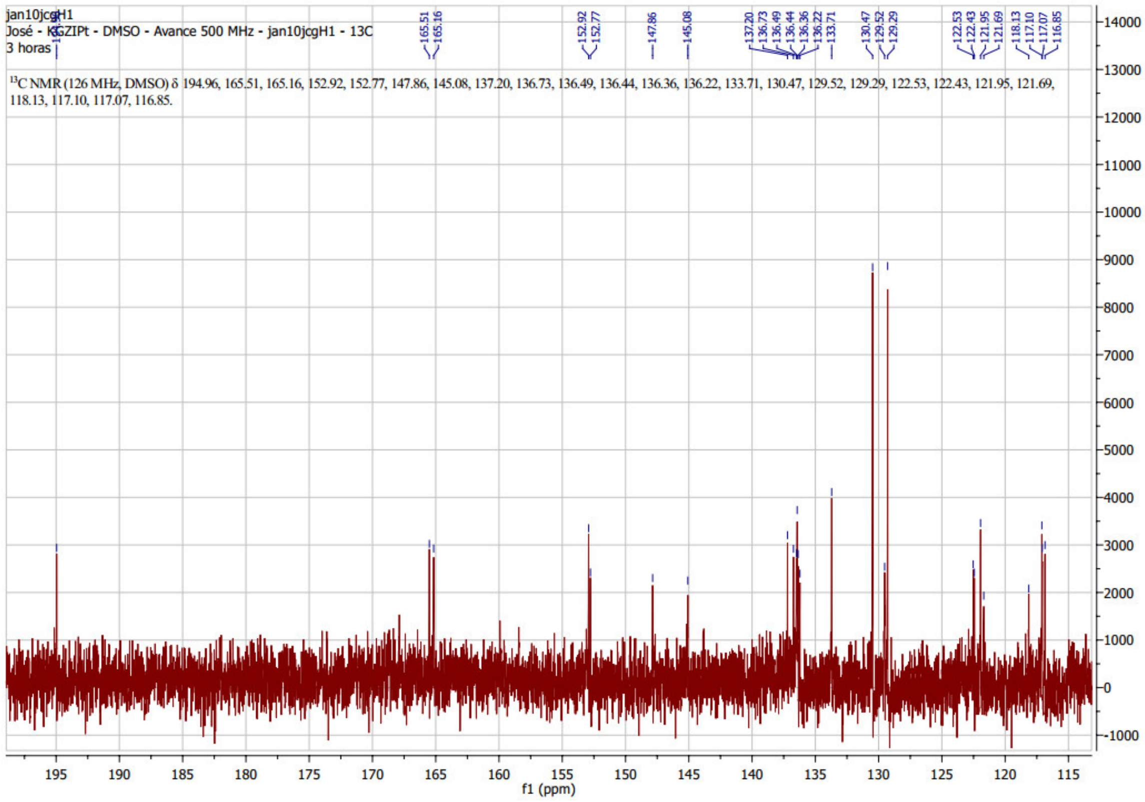

Figure S13. The ^{13}C NMR spectrum of the [Pt(sal-3,4-ben)] solution in DMSO-d_6 (33 mg mL^{-1}).

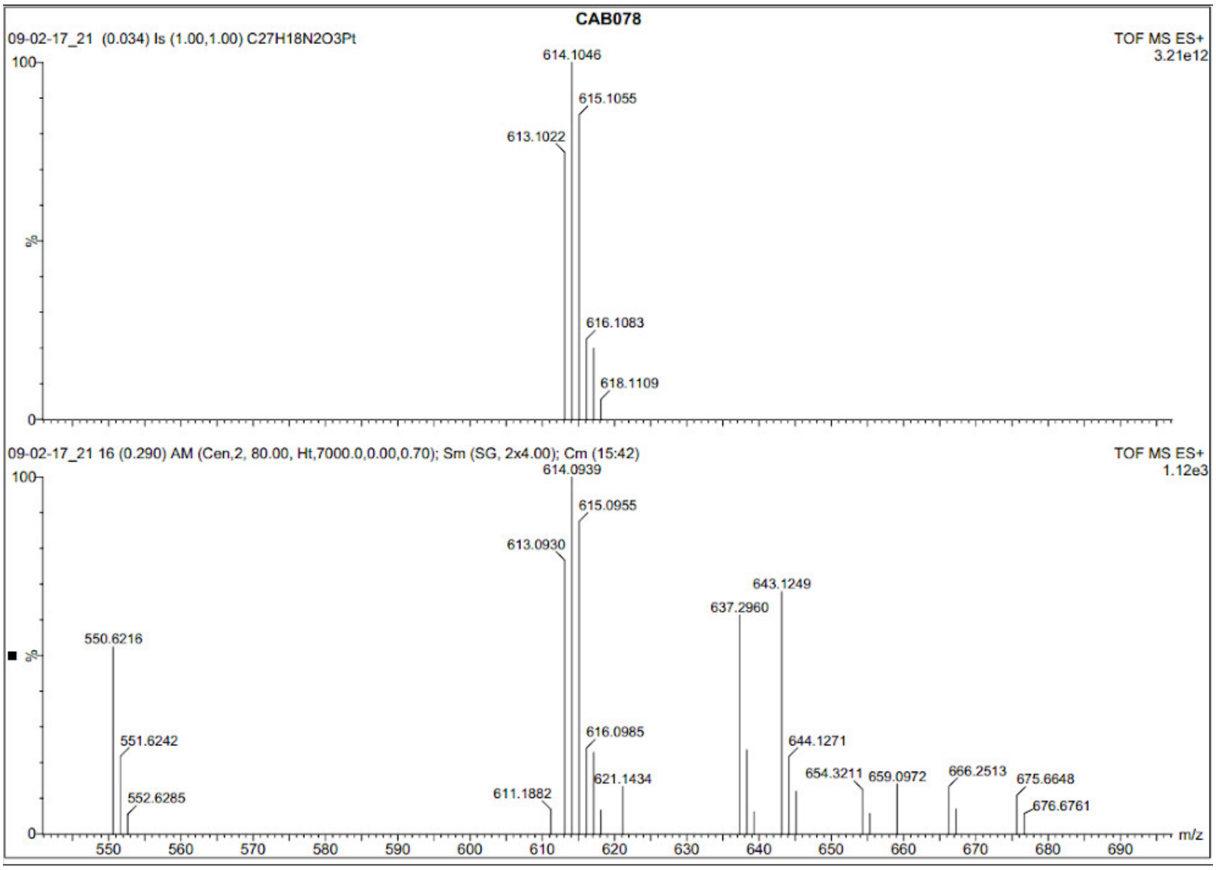

Figure S14. The TOFHR-MS spectra of [Pt(sal-3,4-ben)] in the positive mode.

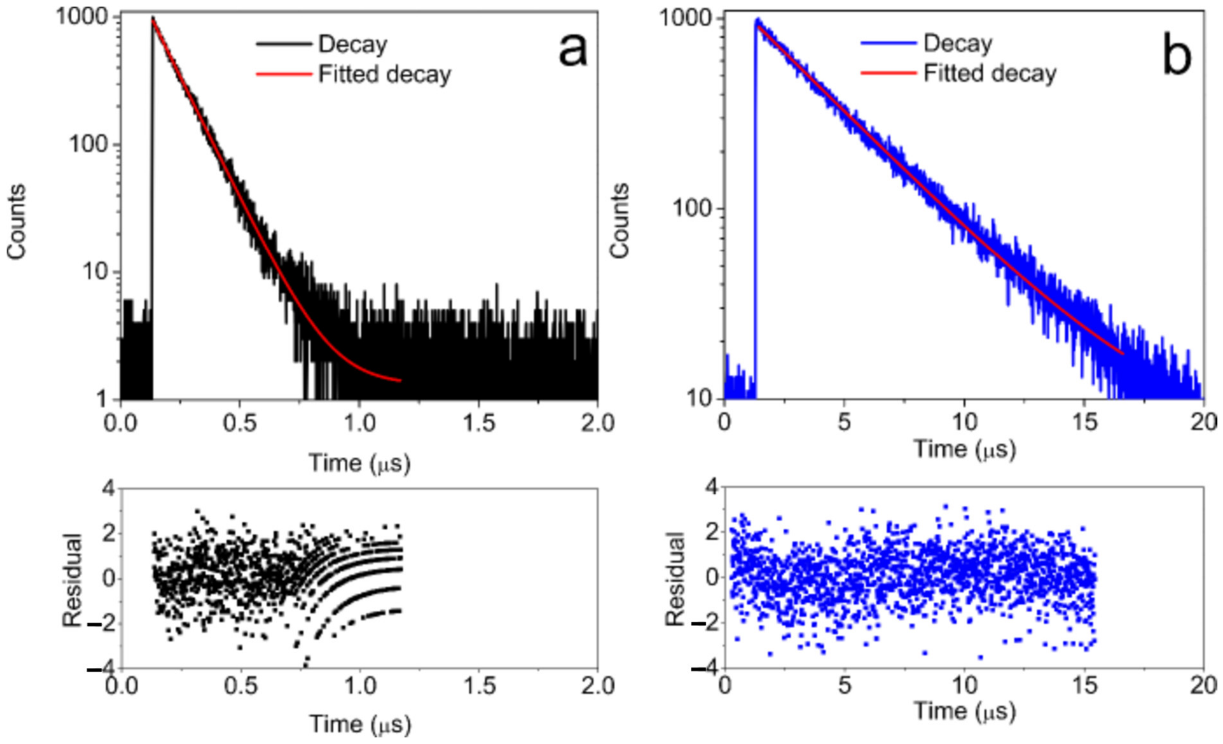

Figure S15. Phosphorescence decays of [Pt(salophen)] (**a**) λ_{exc} = 375 nm; (**b**) λ_{PL} = 621 nm in the THF solution (10 µmol L^{-1}) under N_2(g)- and air-saturated conditions.

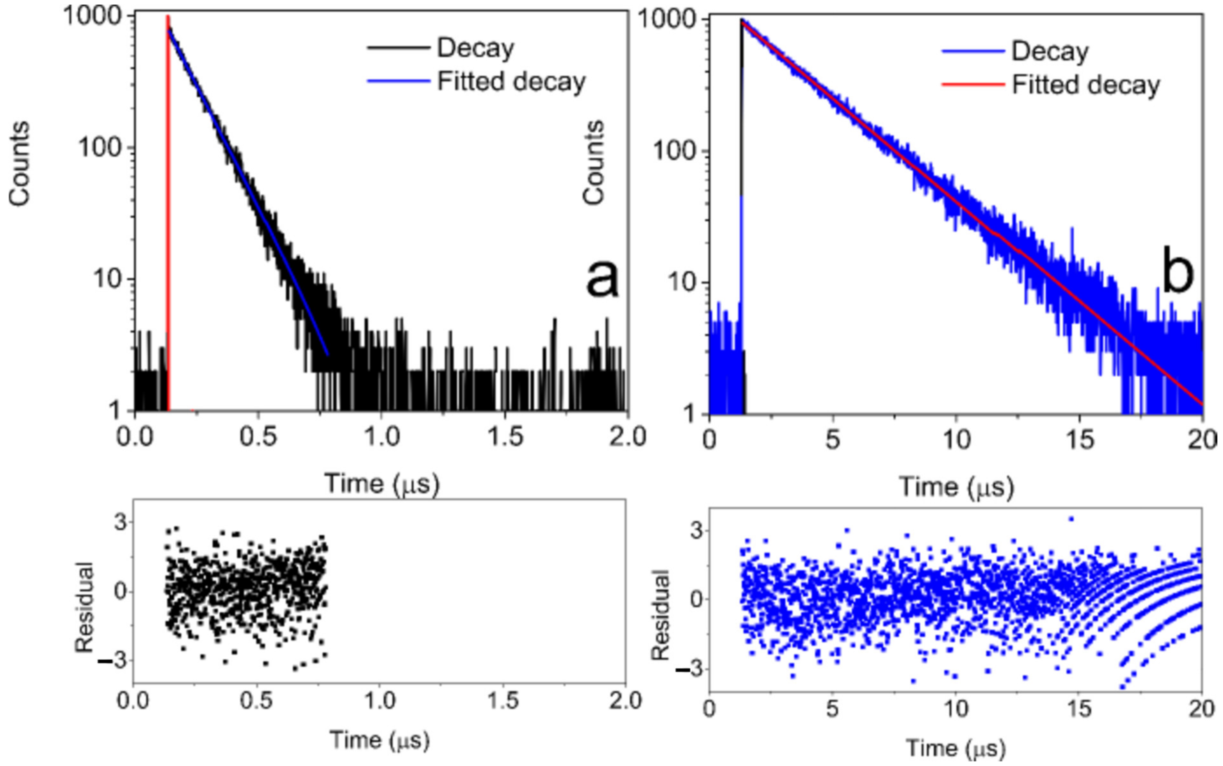

Figure S16. Phosphorescence decays of [Pt(sal-3,4-ben)] (**a**) λ_{exc} = 375 nm; (**b**) λ_{PL} = 637 nm in the THF solution (10 µmol L^{-1}) under N_2(g)- and air-saturated conditions.

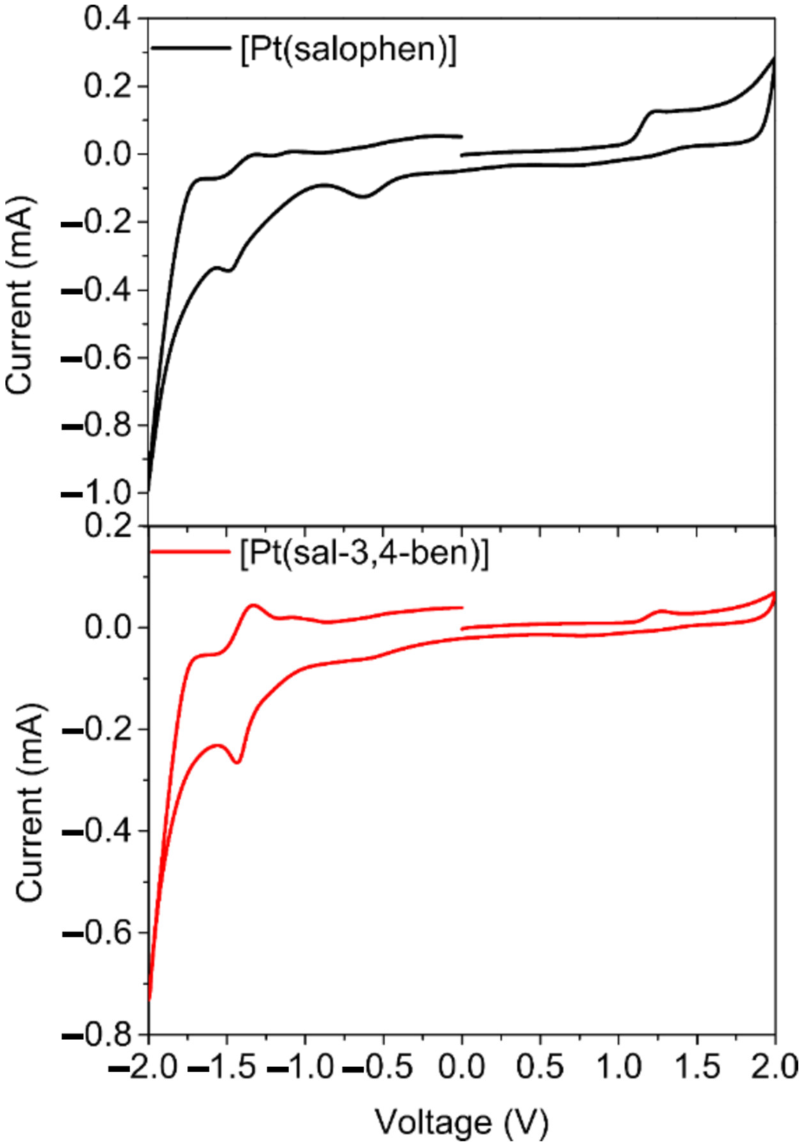

Figure S17. Cyclic voltammograms of [Pt(salophen)] and [Pt(sal-3,4-ben)], measured in ACN solution (1×10^{-4} mol L^{-1}).

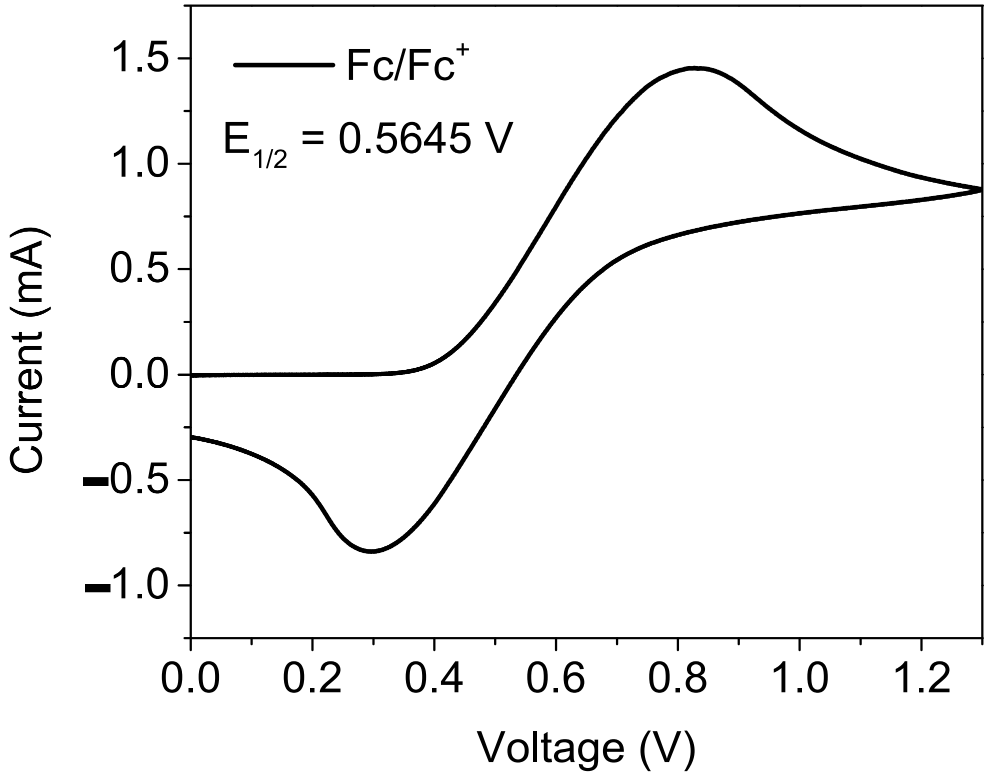

Figure S18. The Fc/Fc^+ internal standard cyclic voltammogram, measured in ACN solution, and its half-wave potential ($E_{1/2}$ = 0.5645 V).

5. Electroluminescent Diode Fabrication

5.1. WOLEDs

ITO substrates (25 Ω/cm² from Delta Technologies) were cleaned with Extran® neutral detergent, deionized water, 2-propanol, and acetone in an ultrasonic bath for 15 min each. The substrates were dried on a hot plate at 130 °C for 10 min and, subsequently, treated with UV ozone for 30 min. Over this substrate, a 30-nm-thick layer of PEDOT:PSS was spin-coated (5000 rpm for 60 s) and annealed for 30 min at 110 °C. Next, 70 nm of the HTL/EBL (PVK), followed by the active layers at ≈ 25 nm of thickness, composed of PFO:Pt(II) in a THF solution, were spin-cast under an Ar(g) atmosphere. Finally, calcium (30 nm) and aluminum (100 nm) layers were deposited via thermal evaporation under a vacuum of 10^{-6} mbar. The concentration of the Pt(II) compounds in the PFO films was at 0.1, 0.5, and 1.0% mol/mol ratio. The final OLED structure is ITO|PEDOT:PSS (30 nm)|PVK (70 nm)|PFO:Pt(II) (25 nm)|Ca (30 nm)|Al (100 nm) (Figure S16).

The OLEDs, held in an oxygen-free sample holder, were characterized in terms of their current density vs. voltage measurements (*J*–*V*) using a 2400 Keithley SourceMeter. Electroluminescence (EL) spectra were acquired using a USB2000+ Ocean Optics diode array spectrometer. The luminance (L) and the CIE 1931 chromaticity coordinates were obtained using a Konica Minolta CS-100A luminance meter with a close-up lens (No. 110, ø = 40.5 mm, 10 to 20 cm). All OLED figures of merit (current efficiency—η_C, power efficiency—η_P, and external quantum efficiency—EQE) were calculated using homemade software developed by Professor Luiz Pereira, considering the Lambertian emissions from the devices.

5.2. Single-carrier devices

P- and n-type diodes were assembled using the following architecture: ITO|PEDOT:PSS|PFO:Pt(II) complex (0.1 and 0.5%)|PEDOT:PSS|Al and ITO|LiF| PFO:Pt(II) complex (0.1 and 0.5%)| |LiF|Al for the only hole and electron transport, respectively. The

conditions for film deposition, the thermal evaporation of LiF and Al, and the JxV measurements were the same as those used for the electroluminescent diodes.

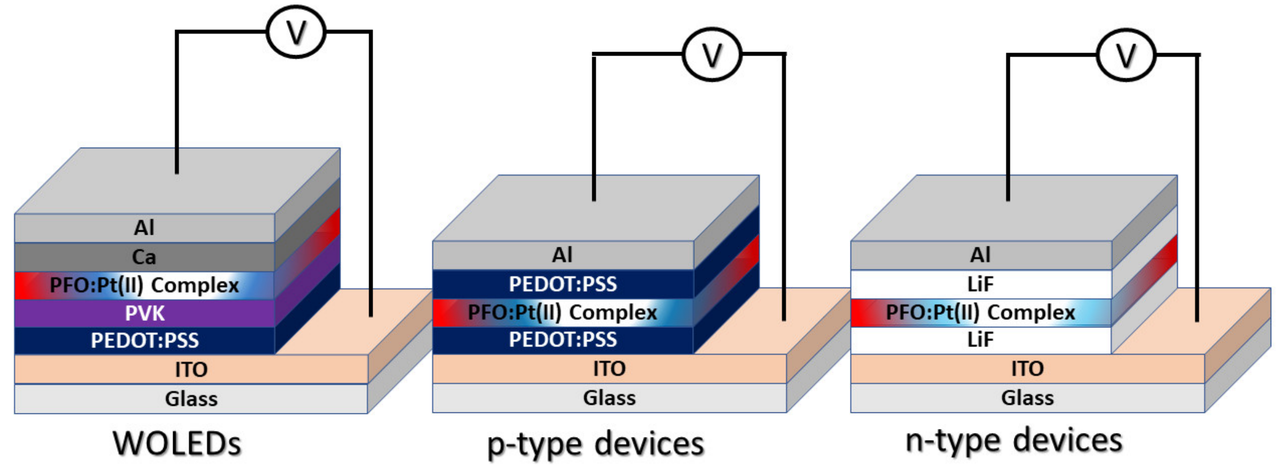

Figure S19. PFO:Pt(II)salicylidenes **s**olution-processed WOLEDs, p- and n-type devices.

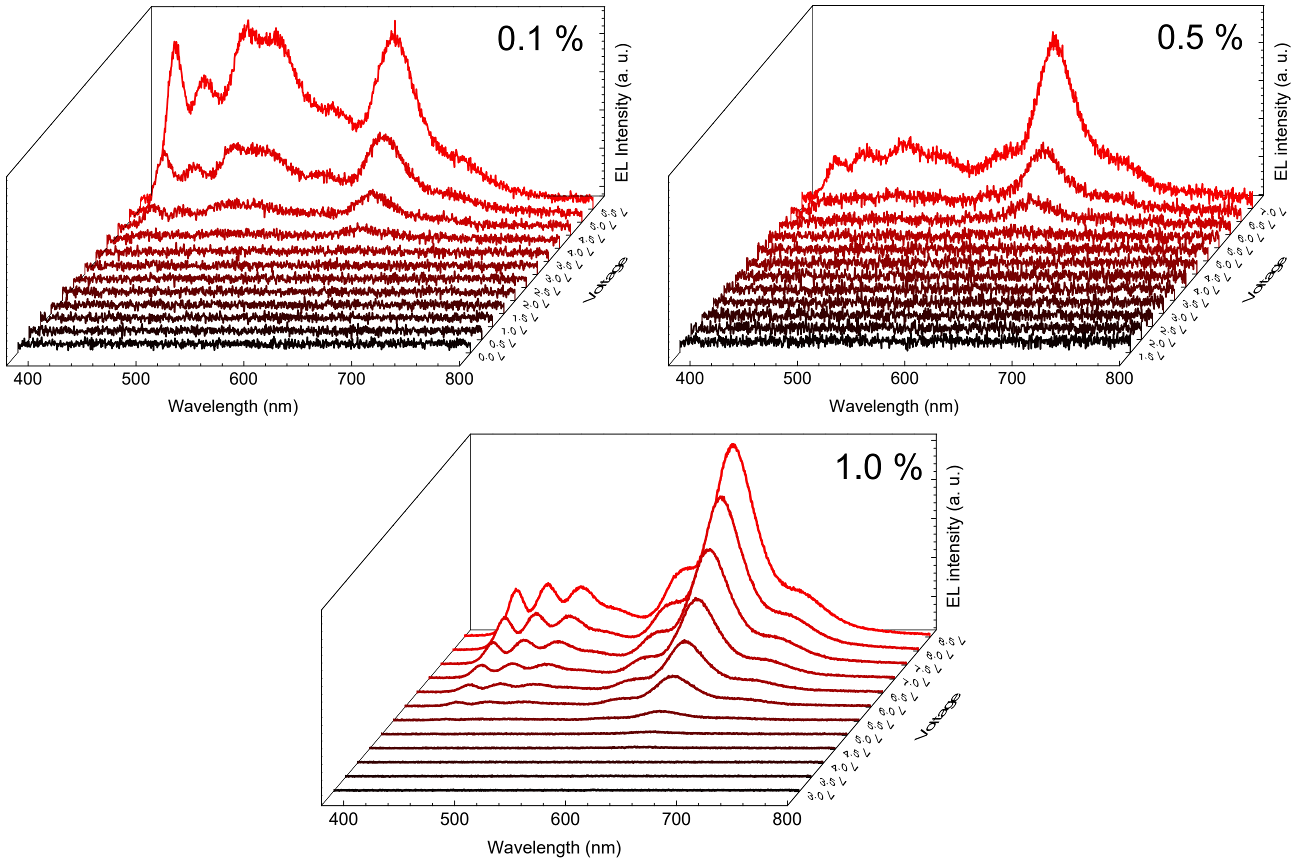

Figure S20. Dependence of the ITO|PEDOT:PSS|PVK|PFO:Pt(salophen) 0.1, 0.5, and 1.0% (mol/mol)|Ca|Al WOLEDs electroluminescence spectra, with the applied bias.

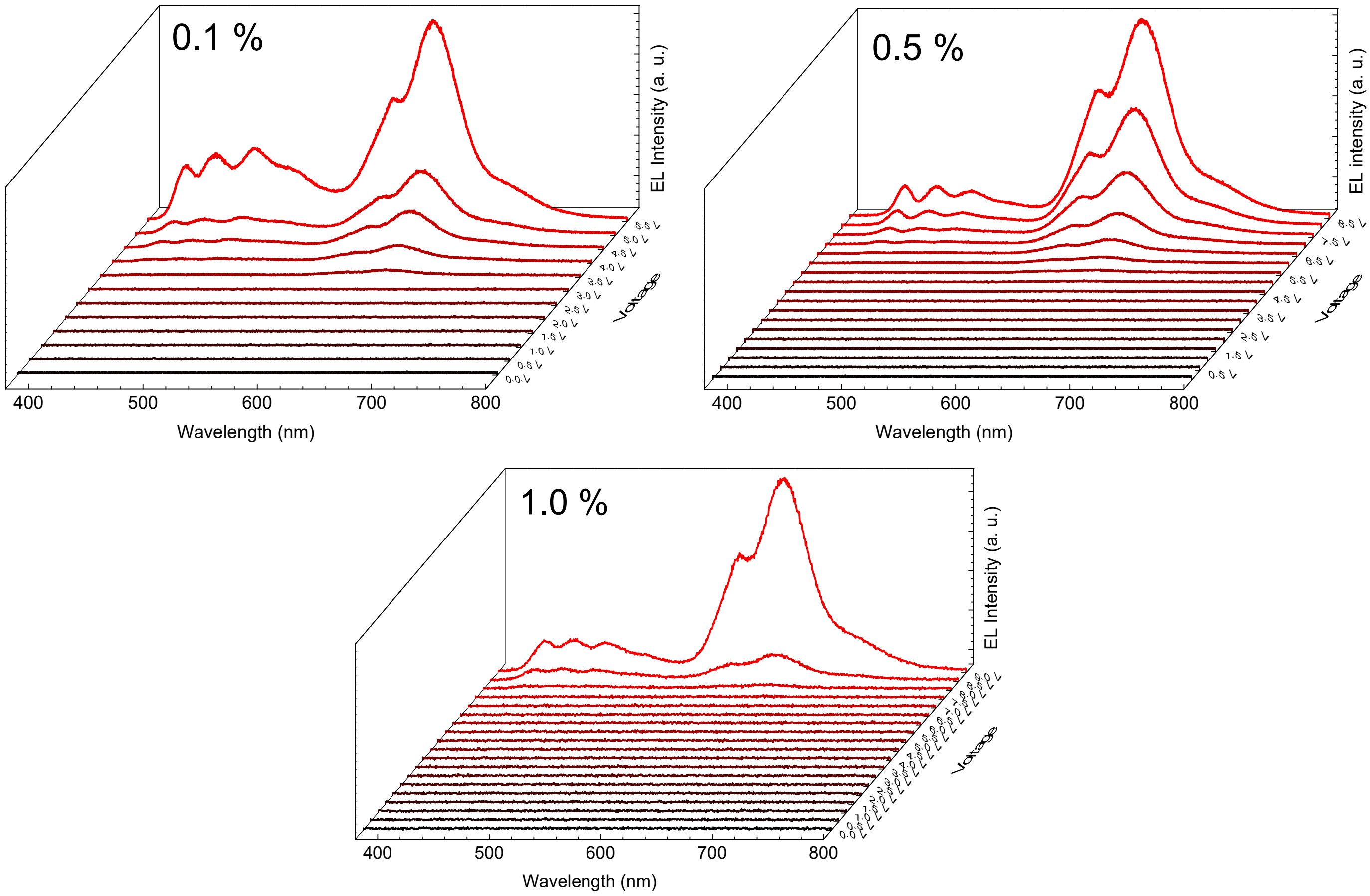

Figure S21. Dependence of the ITO | PEDOT:PSS | PVK | PFO:Pt(sal-3,4-ben) 0.1, 0.5 and 1.0% (mol/mol) | Ca | Al WOLEDs electroluminescence spectra, with the applied bias.

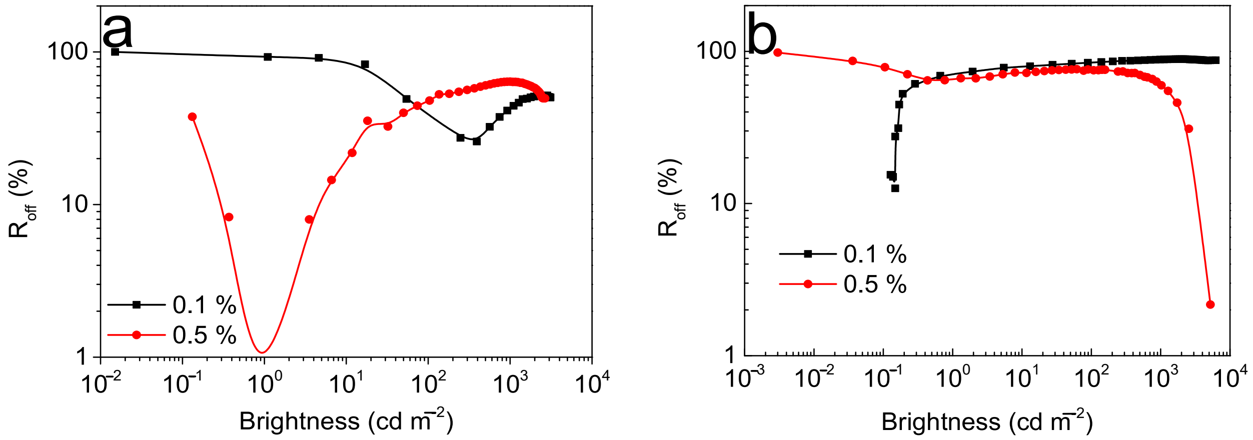

Figure S22. EQE roll-off during working brightness in PFO-based WOLEDs assembled with 0.1 (black curves) and 0.5% (reference curves) of [Pt(salophen)] (**a**) and [Pt(sal-3,4-ben)] (**b**).

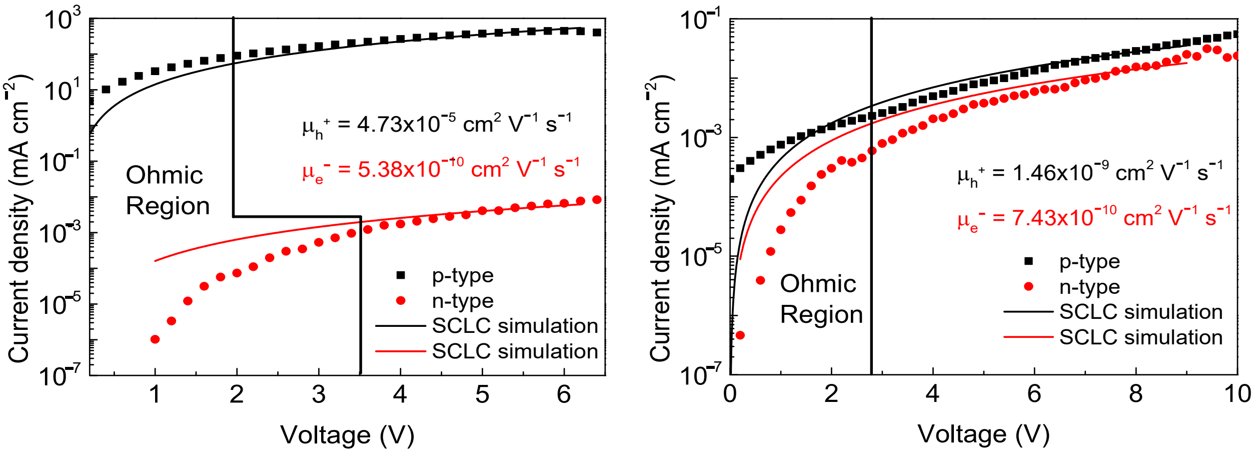

0.1% 0.5%

Figure S23. Current density vs. the voltage curves of hole- (black squares) and electron-only (red circles) devices for 0.1% and 0.5% of the [Pt(sal-3,4-ben)]-based WOLEDs. Straight lines represent the best curve simulation, according to the Mott–Gurney SCLC model.

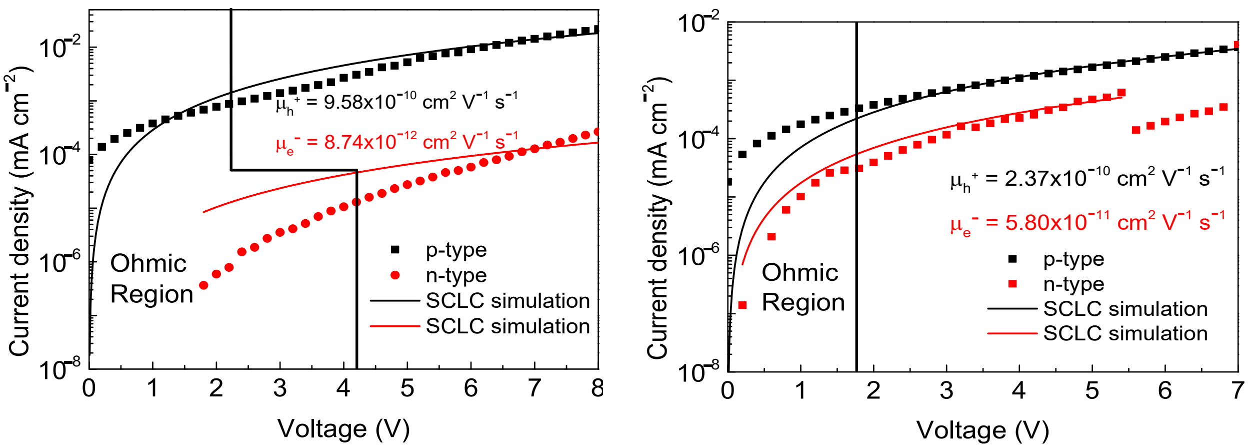

0.1% 0.5%

Figure S24. Current density vs. the voltage curves of hole- (black squares) and electron-only (red circles) devices for 0.1% and 0.5% of the Pt(salophen)-based WOLEDs. Straight lines represent the best curve simulation, according to the Mott–Gurney SCLC model.

Table S3. The optical-electronic parameters of OLEDs with Pt(II) complexes as a guest, where V_{on} is the turn-on voltage (V), L is the maximum luminance (cd m^{-2}), J is the current density (mA cm^{-2}), η is the current efficiency (cd A^{-1}), and EQE (%). EML stands for the emissive layer.

Deposition Technique	Architecture	V_{on}	L	η_{curr}	EQE	Reference
Solution-Processed	ITO/PEDOT:PSS/EML/TPBi/Ba/Al	7.2–9.8	-	-	0.88–2.44	[14]
Solution-Processed	ITO/PEDOT:PSS/EML/TPBi/LiF/Al	4.84–5.74	1018–3500	1.43–2.83	1.32–2.15	[15]
Solution-Processed	ITO/PEDOT:PSS/EML/BCP/LiF/Al	6–9	708–12,000	9.55–12.11	3.20	[16]
Solution-Processed	ITO/PEDOT:PSS/EML/DPEPO/TPBi/LiF/Al	5–6	800–12,600	3.17–39.85	4.72–15.78	[17]
Thermally Evaporated	ITO/NPB/mCP/EML/TPBi/LiF/Al	6.4–8.4	84–1870	0.14–1.12	0.11–0.54	[18]
Thermally Evaporated	ITO/HATCN/BPBPA/TAPC/EML/DPEPO/TmPyPB/LiF/Al	3.4–3.5	5746–13,597	27.30–30.80	11.3–14.7	[19]
Thermally Evaporated	ITO/NPB/EML/BPhen/Liq/Al Or ITO/NPB/EML/BCP/Alq3/Liq/Al	3–4	2000–10,000	6.06–8.03	3.38–4.84	[20]
Thermally Evaporated	ITO/NPB/EML/BCP/Alq3/LiF/Al	2.8–3.8	4500–23,000	2.80–31.00	0.47–11.00	[21]
Thermally Evaporated	ITO/TAPC/TCTA/EML/DPEPO/TPBi /LiF/Al	~4.0	~1000	-	8.00	[22]
Thermally Evaporated	ITO/15% MoO3:85% CzSi/CzSi/EML/TSPO1/TPBi/LiF/Al	~4.6	~100	3.20	3.20	[23]
Solution-Processed	ITO/PEDOT:PSS/PVK/EML/Ca/Al	3.2	6224	12.1	15.3	This work

References

1. Barboza, C.A.; Germino, J.C.; Santana, A.M.; Quites, F.J.; Vazquez, P.A.M.; Atvars, T.D.Z. Structural correlations between luminescent properties and excited state internal proton transfer in some zinc(II) N,N′-bis(salicylidenes). *J. Phys. Chem. C* **2015**, *119*, 6152–6163. https://doi.org/10.1021/jp510476h.
2. Vivas, M.G.; Germino, J.C.; Barboza, C.A.; Simoni, D.D.A.; Vazquez, P.A.M.; De Boni, L.; Atvars, T.D.Z.; Mendonça, C.R. Revealing the Dynamic of Excited State Proton Transfer of a π-Conjugated Salicylidene Compound: An Experimental and Theoretical Study. *J. Phys. Chem. C* **2016**, *121*, 1283–1290. https://doi.org/10.1021/acs.jpcc.6b06366.
3. Zhou, L.; Kwong, C.L.; Kwok, C.C.; Cheng, G.; Zhang, H.; Che, C.M. Efficient red electroluminescent devices with sterically hindered phosphorescent platinum(II) Schiff base complexes and iridium complex codopant. *Chem.-Asian J.* **2014**, *9*, 2984–2994. https://doi.org/10.1002/asia.201402618.
4. Bruker APEX2, version 2010.3-0, Bruker (2010). Bruker AXS Inc **2010**, Madison, WI, USA.
5. Sheldrick, G.M. A short history of SHELX. *Acta Cryst. Sect A Found Cryst.* **2007**, *64*, 112–122. https://doi.org/10.1107/S0108767307043930.
6. Sheldrick, G.M. Crystal structure refinement with *SHELXL*. *Acta Cryst. Sect C Struct Chem* **2015**, *71*, 3–8. https://doi.org/10.1107/S2053229614024218.
7. Macrae, C.F.; Edgington, P.R.; McCabe, P.; Pidcock, E.; Shields, G.P.; Taylor R., Streek J.v.d. Mercury: Visualization and analysis of crystal structures. *J Appl Cryst.* **2006**, *39*, 453–457. https://doi.org/10.1107/S002188980600731X.
8. Brouwer, A.M. Standards for Photoluminescence Quantum Yield Measurements in Solution (IUPAC Technical Report). *Pure Appl. Chem.* **2011**, *83*, 2213–2228. https://doi:10.1351/PAC-REP-10-09-31.
9. Cardona, C.M.; Li, W.; Kaifer, A.E.; Stockdale, D.; Bazan, G.C. Electrochemical Considerations for Determining Absolute Frontier Orbital Energy Levels of Conjugated Polymers for Solar Cell Applications. *Adv. Mater.* **2011**, *23*, 2367–2371. https://doi:10.1002/adma.201004554.

10. Adamo, C.; Barone, V. Toward Reliable Density Functional Methods without Adjustable Parameters: The PBE0 Model. *J. Chem. Phys.* **1999**, *110*, 6158–6170. https://doi:10.1063/1.478522.
11. Bühl, M.; Reimann, C.; Pantazis, D.A.; Bredow, T.; Neese, F. Geometries of Third-Row Transition-Metal Complexes from Density-Functional Theory. *J. Chem. Theory Comput.* **2008**, *4*, 1449–1459. https://doi:10.1021/ct800172j.
12. Pantazis, D.A.; Chen, X.Y.; Landis, C.R.; Neese, F. All-Electron Scalar Relativistic Basis Sets for Third-Row Transition Metal Atoms. *J. Chem. Theory Comput.* **2008**, *4*, 908–919. https://doi:10.1021/ct800047t.
13. Neese, F. Software Update: The ORCA Program System, Version 4.0. *Wiley Interdiscip. Rev. Comput. Mol. Sci.* **2018**, *8*, 1–6. https://doi:10.1002/wcms.1327.
14. Zhang, Y.; Yin, Z.; Meng, F.; Yu, J.; You, C.; Yang, S.; Tan, H.; Zhu, W.; Su, S. Tetradentate Pt(II) 3,6-Substitued Salophen Complexes: Synthesis and Tuning Emission from Deep-Red to near Infrared by Appending Donor-Acceptor Framework. *Org. Electron.* **2017**, *50*, 317–324. https://doi:10.1016/J.ORGEL.2017.08.006.
15. Jiang, Z.; Wang, J.; Gao, T.; Ma, J.; Liu, Z.; Chen, R. Rational Design of Axially Chiral Platinabinaphthalenes with Aggregation-Induced Emission for Red Circularly Polarized Phosphorescent Organic Light-Emitting Diodes. *ACS Appl. Mater. Interfaces* **2020**, *12*, 9520–9527. https://doi:10.1021/acsami.9b20568.
16. Kim, T.-H.; Lee, H.K.; Park, O.O.; Chin, B.D.; Lee, S.-H.; Kim, J.K. White-Light-Emitting Diodes Based on Iridium Complexes via Efficient Energy Transfer from a Conjugated Polymer. *Adv. Funct. Mater.* **2006**, *16*, 611–617. https://doi:10.1002/adfm.200500621.
17. Suo, X.; Nie, C.; Liu, W.; Zhang, Y.; Shen, Y.; Bian, H.; Cheng, G. Red phosphorescent binuclear Pt(<scp>ii</scp>) complexes incorporating bis(diphenylphorothioyl)amide ligands: Synthesis, photophysical properties and application in solution processable OLEDs. *J. Mater. Chem. C* **2021**, *9*, 9505–9514. https://doi.org/10.1039/D1TC02087K.
18. Zhang, J.; Zhu, X.; Zhong, A.; Jia, W.; Wu, F.; Li, D.; Tong, H.; Wu, C.; Tang, W.; Zhang, P.; et al. New Platinum(II) One-Armed Schiff Base Complexes for Blue and Orange PHOLEDs Applications. *Org. Electron.* **2017**, *42*, 153–162. https://doi:10.1016/J.ORGEL.2016.12.024.
19. Li, G.; Zhao, X.; Fleetham, T.; Chen, Q.; Zhan, F.; Zheng, J.; Yang, Y.F.; Lou, W.; Yang, Y.; Fang, K.; et al. Tetradentate Platinum(II) Complexes for Highly Efficient Phosphorescent Emitters and Sky Blue OLEDs. *Chem. Mater.* **2020**, *32*, 537–548. https://doi:10.1021/acs.chemmater.9b04263.
20. Kang, S.K.; Hwang, N.; Pak, S.; Kim, Y.K.; Yoon, S.S. Platinum(II) Complexes Based on Phenylbenzoazole-Derived Ligands for Phosphorescent Organic Light-Emitting Diodes. *J. Nanosci. Nanotechnol.* **2019**, *20*, 589–593. https://doi:10.1166/jnn.2020.17244.
21. Che, C.-M.; Chan, S.-C.; Xiang, H.-F.; Chan, M.C.W.; Liu, Y.; Wang, Y. Tetradentate Schiff Base Platinum(II) Complexes as New Class of Phosphorescent Materials for High-Efficiency and White-Light Electroluminescent DevicesElectronic Supplementary Information (ESI) Available: Synthesis and Spectroscopic, Thermal (TGA), *Photop. Chem. Commun.* **2004**, *224*, 1484–1485. https://doi:10.1039/b402318h.
22. Bullock, J.D.; Salehi, A.; Zeman, C.J.; Abboud, K.A.; So, F.; Schanze, K.S. In Search of Deeper Blues: Trans-N-Heterocyclic Carbene Platinum Phenylacetylide as a Dopant for Phosphorescent OLEDs. *ACS Appl. Mater. Interfaces* **2017**, *9*, 41111–41114. https://doi:10.1021/acsami.7b12107.
23. He, R.; Xu, Z.; Valandro, S.; Arman, H.D.; Xue, J.; Schanze, K.S. High-Purity and Saturated Deep-Blue Luminescence from trans -NHC Platinum(II) Butadiyne Complexes: Properties and Organic Light Emitting Diode Application. *ACS Appl. Mater. Interfaces* **2021**, *13*, 5327–5337. https://doi.org/10.1021/acsami.0c21193.
